# Overall similarity and consistency assessment scores are not sufficiently accurate for predicting discrepancy between direct and indirect comparison estimates

**DOI:** 10.1016/j.jclinepi.2012.06.022

**Published:** 2013-02

**Authors:** Tengbin Xiong, Sheetal Parekh-Bhurke, Yoon K. Loke, Asmaa Abdelhamid, Alex J. Sutton, Alison J. Eastwood, Richard Holland, Yen-Fu Chen, Tanya Walsh, Anne-Marie Glenny, Fujian Song

**Affiliations:** a Norwich Medical School, University of East Anglia, Norwich Research Park, Norwich, Norfolk, NR4 7TJ, UK; b Department of Oncology, University of Cambridge, Cambridge, UK; c NIHR Trials & Studies Coordinating Centre, University of Southampton, Southampton, UK; d Department of Health Science, University of Leicester, Leicester, UK; e Centre for Reviews and Dissemination, University of York, York, UK; f Department of Public Health, Epidemiology and Biostatistics, University of Birmingham, Birmingham, UK; g School of Dentistry, University of Manchester, Manchester, UK

**Keywords:** Indirect treatment comparison, Mixed treatment comparison, Multiple treatment meta-analysis, Network meta-analysis, Trial similarity, Evidence consistency

## Abstract

**Objectives:**

Indirect comparison methods have been increasingly used to assess the effectiveness of different interventions comparatively. This study evaluated a Trial Similarity and Evidence Consistency Assessment (TSECA) framework for assessing key assumptions underlying the validity of indirect comparisons.

**Study Design and Setting:**

We applied the TSECA framework to 94 Cochrane Systematic Reviews that provided data to compare two interventions by both direct and indirect comparisons. Using the TSECA framework, two reviewers independently assessed and scored trial similarity and evidence consistency. A detailed case study provided further insight into the usefulness and limitations of the framework proposed.

**Results:**

Trial similarity and evidence consistency scores obtained using the assessment framework were not associated with statistically significant inconsistency between direct and indirect estimates. The case study illustrated that the assessment framework could be used to identify potentially important differences in participants, interventions, and outcome measures between different sets of trials in the indirect comparison.

**Conclusion:**

Although the overall trial similarity and evidence consistency scores are unlikely to be sufficiently accurate for predicting inconsistency between direct and indirect estimates, the assessment framework proposed in this study can be a useful tool for identifying between-trial differences that may threaten the validity of indirect treatment comparisons.

## 1 Introduction

**What is new?**

**Key findings**
• Trial similarity and evidence consistency scores obtained using the assessment framework were not associated with statistically significant inconsistency between direct and indirect estimates.

**What this adds to what was known?**
• Trial similarity and evidence consistency should be appropriately assessed in any indirect or mixed treatment comparisons. This study evaluated a Trial Similarity and Evidence Consistency Assessment framework for assessing key assumptions underlying the validity of indirect comparisons.

**What is the implication and what should change now?**
• The overall trial similarity and evidence consistency scores are unlikely to be sufficiently accurate for predicting inconsistency between direct and indirect estimates. However, the assessment framework proposed in this study can be a useful tool for identifying between-trial differences that may threaten the validity of indirect treatment comparisons.

The use of indirect comparisons for evaluating comparative effectiveness of different treatments has greatly increased [1,2]. Simple and complex statistical methods have been developed to make indirect treatment comparisons so that the strength of randomized controlled trials may be partially preserved [3–6]. However, the validity of indirect comparison methods is still controversial [7–9]. A recent study found that a significant inconsistency between direct and indirect evidences might be more prevalent than those previously estimated [10].

The validity of indirect treatment comparisons is dependent on some basic assumptions [1,11]. For the indirect comparison of interventions B and C based on a common comparator A, the similarity assumption requires that the average relative effect estimated by one set of trials (AvB trials) is generalizable to the other set of trials (AvC trials). To combine the results of direct and indirect comparisons (i.e., mixed treatment comparisons), an additional evidence consistency assumption is required, which assumes that the evidence from different sources is sufficiently consistent. Imbalance in the distribution of relative treatment effect modifiers across the trials involved in the indirect and mixed treatment comparisons will violate the similarity and consistency assumptions [1,12].

It has been recommended that the trial similarity and evidence consistency should be appropriately assessed in any indirect or mixed treatment comparisons [1,2]. However, practical methods for assessing clinical similarity and evidence consistency across trials have not been tested. This study aimed to propose and empirically evaluate an assessment framework for assessing key assumptions related to the exchangeability of evidence in indirect comparisons. We hypothesized that the assessment framework could be used to predict the observed discrepancy between direct and indirect comparisons. In this article, we report the overall findings based on a sample of 94 Cochrane Systematic Reviews (CSRs), followed by a detailed case study.

## 2 Methods

The methods for the identification of relevant CSRs have been described in a previous publication [10]. Briefly, we searched the Cochrane Database of Systematic Reviews to identify CSRs that provided data to compare two competing interventions (B and C) by both direct and independent indirect comparisons based on a common comparator (A). The basic characteristics of the 94 CSRs included in this study are shown in Table S1 (see at www.jclinepi.com).

### 2.1 Data extraction

From the included CSRs, one reviewer initially extracted the following data into an evidence table: relevant trials for direct or indirect comparisons, the outcome of interest, and event rates in the common comparator arms (Table S2; see at www.jclinepi.com). Then, two reviewers used the evidence table to extract data from three sets of trials (BvC trials for direct comparison, and AvB and AvC trials for indirect comparison) in each of the included CSR. We estimated the typical ranges of important characteristics of patients (age, sex ratio, and severity); interventions (dosages or intensity); outcome measures; and length of follow-up separately for the BvC, AvB, and AvC trials. A narrative summary was provided about the similarities and differences between these sets of trials.

We relied on study tables presented in CSRs to obtain the data required. The full publication of the trials was used when necessary to check or supplement data retrieved from CSRs. The data extraction was independently conducted in duplicate by the two reviewers. Any disagreement was resolved by discussion and/or by the involvement of a third reviewer. To achieve blinded assessment, the two independent reviewers were not aware of the results of indirect comparisons, and relevant data plots were removed from the CSRs during data extraction.

### 2.2 Similarity and consistency assessment

In the assessment of clinical similarity between trials in indirect comparisons, the first key question we asked was “are there any noticeable differences between the two sets of trials in terms of trial participants, interventions, outcomes measured, and other study level variables”? If so, then we considered the next question: “is the relative effect likely to be different because of the observed differences between AvB and AvC trials”? To facilitate answering these two important but judgemental questions, we designed a Trial Similarity and Evidence Consistency Assessment (TSECA) framework.

Our TSECA framework consists of four components: the aforementioned evidence table, a clinical trial similarity assessment (TSA), a quality similarity assessment (QSA), and an evidence consistency assessment (ECA). The TSA and QSA relate to the validity of adjusted indirect comparisons, whereas the ECA relates to the validity of mixed treatment comparisons.

Using data compiled in the evidence table, two researchers independently assessed the clinical similarity of the two sets of trials (AvB and AvC) in the indirect comparisons. The results of the assessment of clinical similarity were conveyed as a TSA score ranging from one (very low, with severe concerns) to five (very high similarity, with no concern), using the TSA sheet for assessing trial clinical similarity (Table S3; see at www.jclinepi.com). The development of the scales aimed to elicit perceived trial similarity based on the main characteristics of study participants, interventions (including control), and outcome measures, aspects that are recommended for clinical heterogeneity investigation in meta-analysis [13].

The quality of trials was scored from one (very low) to five (very high), according to randomization method, allocation concealment, blinding of patients and outcome assessors, and reported dropouts (Table S4; see at www.jclinepi.com) [14]. The quality scores of multiple trials were weighted by the number of patients in each trial to calculate an average quality score. When a set of trials is more or less biased than another set of trials, the adjusted indirect comparison may underestimate or exaggerate the relative effect [7]. Therefore, the quality similarity (QSA score) was measured by comparing the average quality scores of the two trial sets.

Using a method similar to the TSA described previously, ECA was conducted to examine consistency of trial evidence between direct and indirect estimates. The ECA was also based on overall characteristics of trial participants, interventions compared, and outcomes measured (Table S5; see at www.jclinepi.com). For example, interventions B and C in the two sets of indirect comparison trials (i.e., AvB and AvC trials) should be consistent with interventions B and C in the head-to-head comparison trials (BvC trials). The results of ECA were also scored from one (very low) to five (very high consistency). In principle, the total ECA score should be equal to or lower than the TSA score.

The assessment of trial similarity and evidence consistency is inevitably a subjective process. The assessment was conducted by two reviewers independently and any disagreement was discussed at weekly project meetings with the involvement of other team members. However, the disagreement in the assessment scores between the two assessors was allowed to remain even after the discussion. A simple average score was calculated when the final scores were different between the two assessors.

### 2.3 Data analysis methods

The interassessor reliability was investigated using the Bland–Altman method [15]. The construct validity of the assessment of trial similarity was tested by investigating whether similarity scores are associated with the extent of discrepancy between the direct and indirect estimates. We used the method by Bucher et al. [4] to conduct indirect comparisons, and estimated statistical inconsistency between the direct and indirect comparisons. The method used to calculate the quantitative discrepancy between direct and indirect estimates has been previously described [10,16]. It was hypothesized that trial dissimilarity or evidence inconsistency as measured by the TSECA framework may be associated with greater discrepancy between the direct and indirect comparisons.

## 3 Results

### 3.1 Main findings

The results of the similarity and consistency assessments, as well as the main characteristics of the included CSRs, are presented in Table S1 (see at www.jclinepi.com).

The differences between the two assessors were visually distributed around zero (Figure S1; see at www.jclinepi.com). One reviewer tended to give a higher score than another for the clinical similarity (mean difference = 0.29; 95% confidence interval [CI] = 0.17–0.42) and consistency (mean difference = 0.28; 95% CI = 0.16–0.41) of outcome measures (Figure S2; see at www.jclinepi.com). In addition, a higher score was given by the same reviewer to the consistency of interventions. There was no significant difference in the quality similarity score between the two reviewers.

**Fig. 1:**
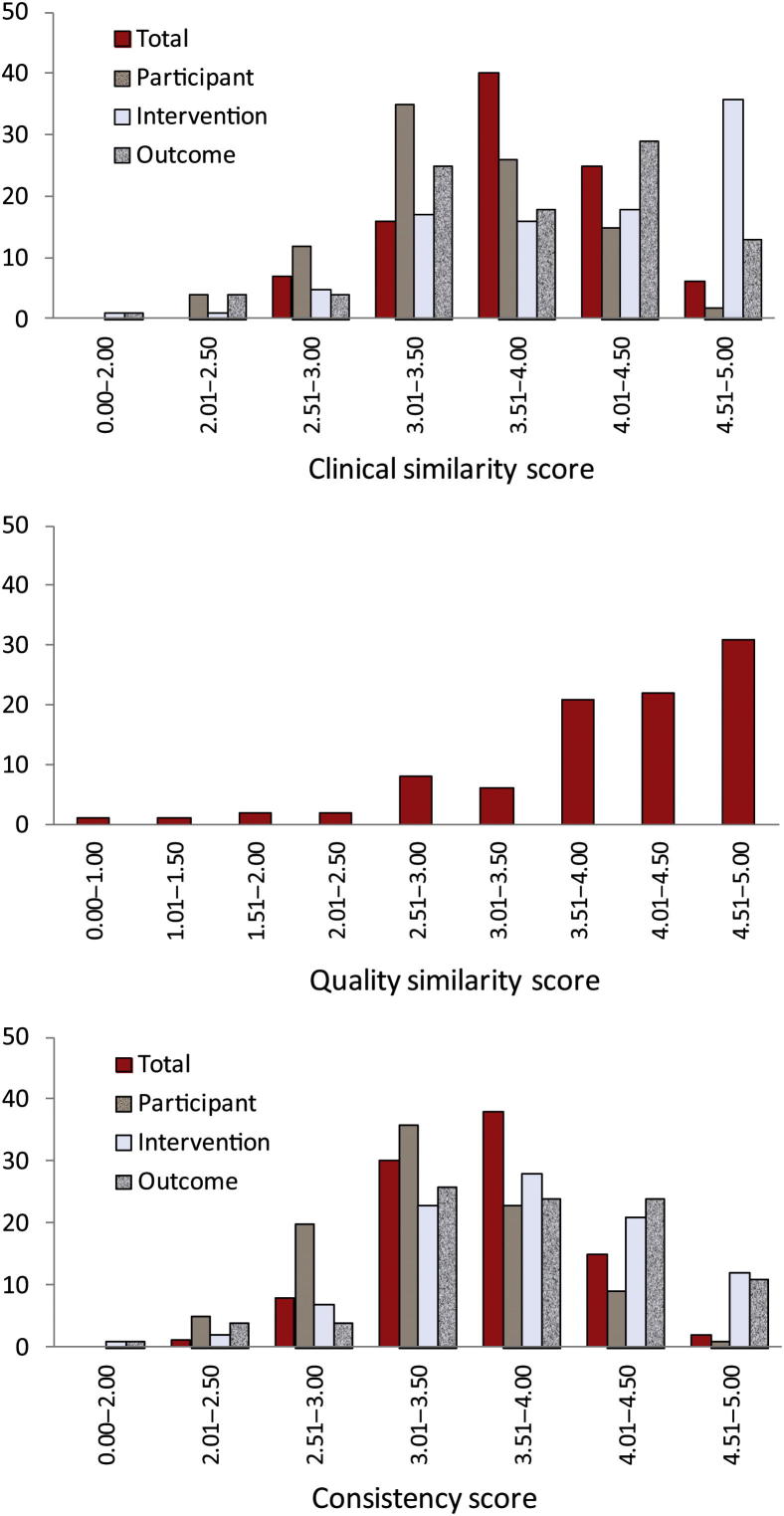
Distribution of similarity and consistency assessment scores.

The overall TSA score was symmetrically distributed (median = 3.83; range = 2.56–4.79; Fig. 1). Of the three clinical components, the participant similarity scores (median = 3.42) were on average lower than the intervention and outcome similarity scores (median = 4.17 and 4.00, respectively). With a median score of 4.24, the distribution of QSA scores is negatively skewed. Note that a high quality similarity score may be achieved if the two sets of trials were similarly good or poor. The distribution of ECA scores is similar to that of TSA scores (Fig. 1).

**Fig. 2:**
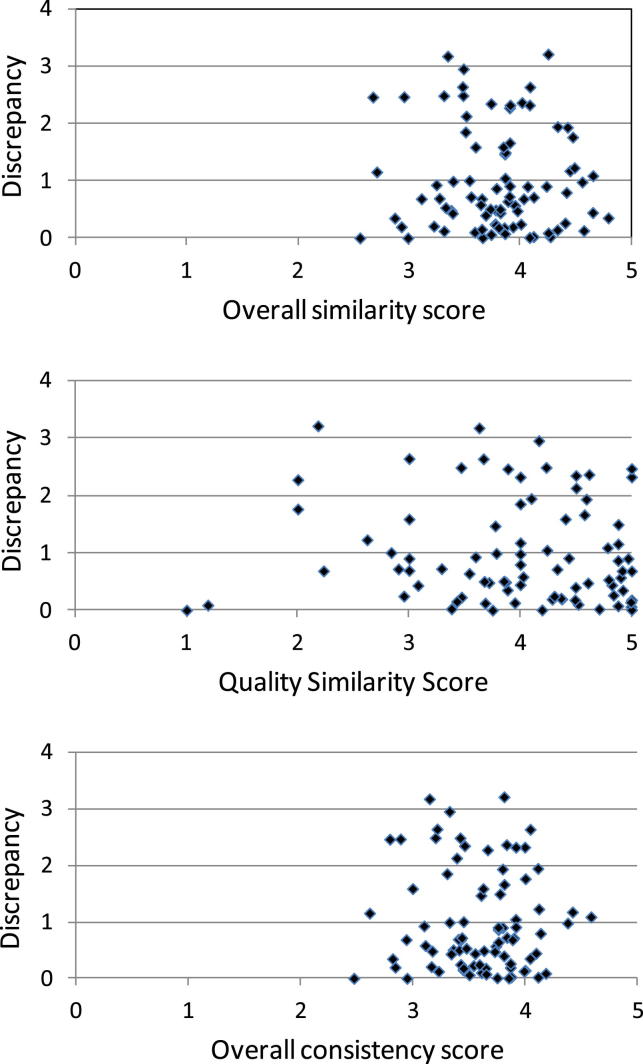
Similarity and consistency scores against absolute discrepancy (in log ratio of odds ratios) between direct and indirect estimates.

Statistically significant discrepancy between direct and indirect estimates (*P* < 0.05) was observed in 16 of the 94 CSRs included. The proportion of significant discrepancy was similar when studies were groups according to similarity or consistency scores (Table 1). There was no association between any assessment scores and the discrepancies between direct and indirect estimates, using data from 83 CSRs in which odds ratio (OR) could be used as the outcome statistic (Fig. 2).

### 3.2 A case study

**Fig. 3:**
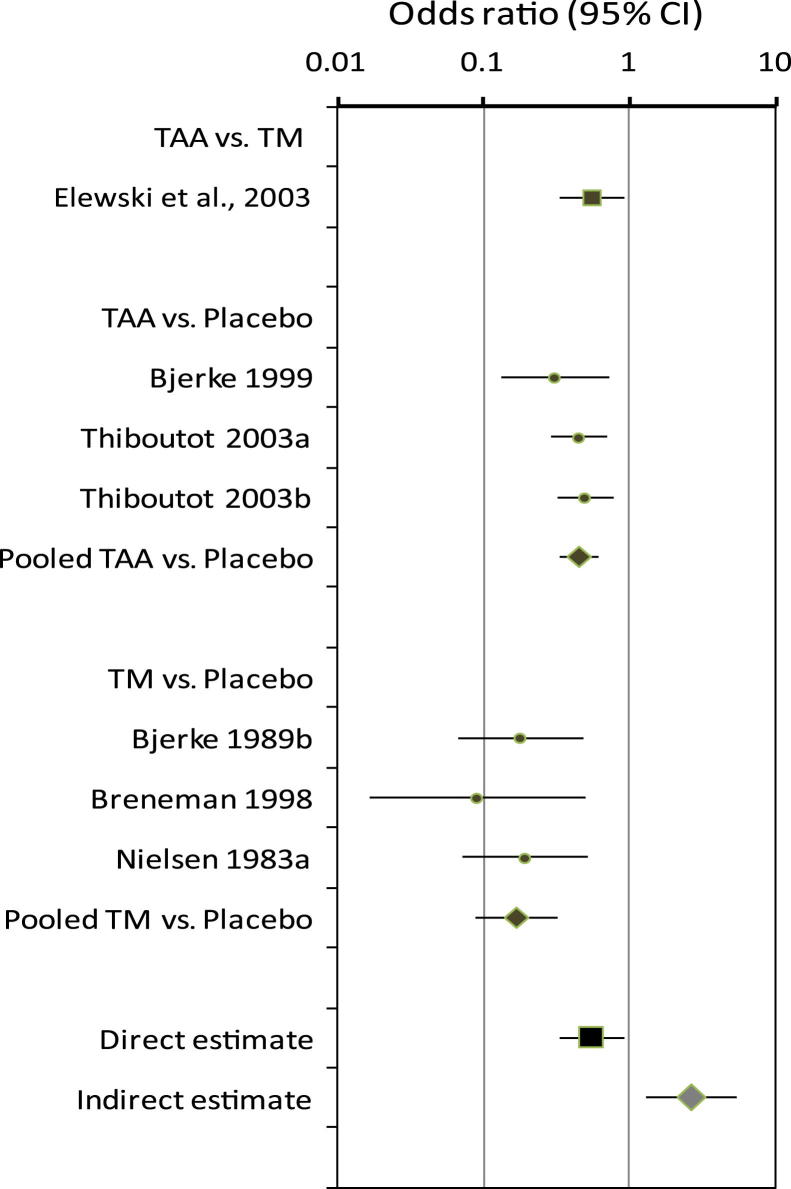
TAA vs. TM for rosacea (outcome: lack of improvement according to physician's global evaluation). TAA, topical azelaic acid; TM, topical metronidazole.

The case study used data from a Cochrane review that compared topical azelaic acid and topical metronidazole in patients with rosacea [17]. A single direct comparison trial found that topical azelaic acid was more effective than topical metronidazole (OR = 0.55; 95% CI = 0.33–0.91) [18]. However, the adjusted indirect comparison based on six trials suggested that azelaic acid was less efficacious than metronidazole (OR = 2.68; 95% CI = 1.31 to 5.46; Fig. 3).

In the assessment of similarity and consistency across trials, we observed some differences between trials in baseline severity, doses and treatment durations, tools used to assess subjective outcomes, and length of follow-up (Table S6–S9; see at www.jclinepi.com). However, the importance of these observed differences were sometimes interpreted differently by the two assessors. The average TSA score was 3.85, the QSA score was 4.41, and the ECA score was 3.63. These overall scores were not different from those of the other cases without significant inconsistency between the direct and indirect estimates, and thus could not explain the striking inconsistency observed in this case.

**Fig. 4:**
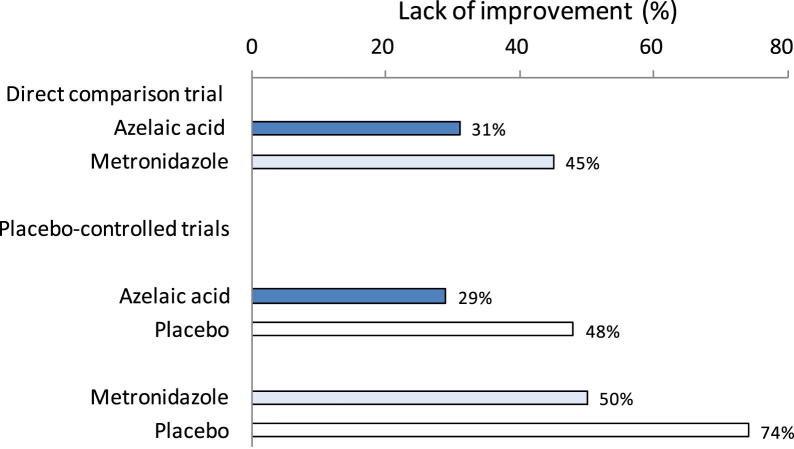
Topical azelaic acid vs. topical metronidazole for rosacea: response by study arms.

Fig 4 shows pooled event rates in single arms of different sets of trials. The pooled event rate in the placebo arm in the azelaic acid trials was considerably lower than that in the metronidazole trials (48% vs. 74%). This difference raises doubt about the similarity of patients between the two sets of trials in the indirect comparison. Patients included in the early metronidazole trials seemed less likely to have spontaneous improvement, as compared with those in the azelaic acid trials. However, patients in the placebo-controlled trials responded similarly to the two active treatments as those in the direct comparison trial (31% vs. 29% to azelaic acid and 45% vs. 50% to metronidazole). Therefore, the difference in the placebo effect between the two sets of trials is likely an important effect modifier.

The direct comparison trial compared a novel, newly developed 15% gel formulation of azelaic acid and 0.75% metronidazole gel [18]. The authors received funding from the manufacturer of the new azelaic acid gel. The newly developed azelaic acid gel in the direct comparison may be different from that used in the placebo-controlled trials. In addition, metronidazole gel was used in the direct comparison trial, whereas metronidazole cream was used in the placebo-controlled trials. However, it is unclear whether these differences in the treatments modified the relative treatment effects.

In summary, we were able to identify some differences in participants, interventions, and outcome measures between trials using the TSECA framework. However, it is difficult to decide the importance of the observed differences in terms of effect modifications. After further detailed consideration, we conclude that the indirect comparison may not be valid in this case owing to the different response to placebo between the two sets of trials in the indirect comparison. In addition, the result from the direct comparison may suffer from the optimum bias that may be present when new drugs are compared with old drugs in direct comparison trials [19].

## 4 Discussion

A recent study based on data from published systematic reviews (including the CSRs used in this study) reported that the inconsistency between direct and indirect comparisons was statistically significant in 14% of the cases, which is more prevalent than those previously observed [10]. Based on theoretical considerations, we have hypothesized that the assessment of trial similarity and evidence consistency may predict the significant inconsistency between direct and indirect comparisons. However, this hypothesis was not confirmed by the findings from this study. We found no relationship between the assessment scores and the discrepancy between the direct and indirect comparisons. This result may be explained by the following reasons:

• Assessors' insufficient knowledge and understanding of clinical topics,
• Inadequate information available from the CSRs and primary studies,
• Inadequate sensitivity and/or specificity of the tools, and
• Difficulties in identifying effect modifiers.

### 4.1 Insufficient understanding of the clinical topics

The two assessors who assessed the similarity and consistency have backgrounds of medical- and health-related training. However, a large number of Cochrane reviews on a wide range of clinical topics were included. For each included case, they had only 2–3 days on average to complete the assessment forms, and to make subjective judgements about trial similarity and evidence consistency. Because of the time restriction and clinical diversities, their understanding of the relevant clinical topics was likely to be superficial or inadequate in many cases. The two assessors were able to identify some possible effect modifiers. For example, they noticed difference in placebo response rate between trials in the case study (Table S7; see at www.jclinepi.com). Clinical experts may help the identification of potential treatment effect modifiers and for the interpretation of observed differences. Further research is required to evaluate the involvement of clinical content specialists in the assessment of trial similarity and evidence consistency.

### 4.2 Inadequate information available

The assessment was based mainly on data extracted from study tables in the included CSRs. Information presented in study tables in some CSRs may not be adequate for an appropriate assessment of trial similarity and evidence consistency. In addition, assessors were blinded to the quantitative results of the relevant meta-analyses to avoid possible bias in the subjective assessment process.

### 4.3 Limitations of the assessment tools

The assessment of similarity and consistency is a process of subjective judgment based mainly on data from study tables in the included CSRs. The difference in overall similarity and consistency scores between the two assessors was statistically significant. Although the two assessors were able to identify similar differences in participants, interventions, and outcomes, they often disagreed on the perceived impact of the observed differences on the effect estimates of relative effectiveness. In some cases, the disagreement remained even after discussion, as is shown in the case study. Therefore, the tools proposed may not be sufficiently reliable.

The assessment tools used may have inadequate sensitivity and specificity. A large number of variables were considered in the assessment of trial similarity and evidence consistency. Difference in only one variable may be sufficient to violate the trial similarity or evidence consistency assumptions. Therefore, an average score by pooling subscores of many variables may conceal the key threat(s) to the validity of indirect comparisons. However, in further exploratory analyses, we again failed to find any clear association between the minimum items-specific assessment scores and the observed inconsistency between direct and indirect estimates.

### 4.4 Difficulties in identifying effect modifiers

The validity of the adjusted indirect comparison depends on the trial similarity in terms of modifiers of relative treatment effects, an issue related to external validity or generalizability of trial results. For an adjusted indirect comparison of BvC to be valid, the result from AvB trials should be generalizable to patients in AvC trials, and the result of AvC trials should be generalizable to patients in AvB trials. If the effect modifiers are distributed unevenly among trials, the generalizability of results between the trials will be problematic.

The term effect modifier and moderator are often considered synonymous. Kraemer et al. [20] defined “moderators of treatment outcomes” as “a pretreatment or baseline variable that identifies subgroups of patients within the population who have different effect size.” More generally, any variables that affect the relative effect of a treatment, or related to external validity, are relevant for the assessment of trial similarity and evidence consistency. Rothwell [21] provided a long list of possible factors that may affect the external validity, including study settings, selection of patients, intervention characteristics, outcome measures, and follow-up.

It is important to note that the same factors may be responsible for heterogeneity in pairwise meta-analysis, invalid adjusted indirect comparison, and inconsistency between direct and indirect estimates [22]. The frequency of treatment–effect modification was estimated to be at least 10%, based on empirical observations of significant heterogeneity in pairwise meta-analyses [23]. The presence of heterogeneity in pairwise meta-analyses may also lead to an imbalance in the distribution of effect modifiers across different sets of trials for indirect and mixed treatment comparisons, which is more likely when the number of studies in a network meta-analysis is small. For example, the inclusion of small numbers of trials is associated with the risk of statistically significant inconsistency between direct and indirect comparisons [10].

Although quantitative methods are available to investigate heterogeneity across studies in meta-analysis and the inconsistency between direct and indirect comparisons, the assessment of trial similarity for adjusted indirect comparison is mainly based on subjective judgment [24]. Some recent studies attempted to improve the validity of indirect and mixed treatment comparisons by adjusting for study-level covariables [25–27]. The meta-regression methods have often limited use because of insufficient number of available studies.

In most circumstances, we may be able to identify some differences in patient characteristics, interventions, and outcomes measured between trials. However, the observed between-trial differences may not necessarily modify relative treatment effects [11]. A predictor of the outcome may have similar effect on patients in the intervention group and patients in the control group so that the relative treatment effect is not affected. Generally, there is a lack of adequate clinical understanding and empirical evidence on plausible effect modifiers, which may be the main cause of the poor predictability of the assessment scores.

### 4.5 Lessons learnt from the case study

We used the case study to demonstrate the actual use of the TSECA framework, and to conduct further detailed, exploratory investigation of trial similarity and evidence consistency. In the case study, the quantitative inconsistency between the direct and indirect estimates could not be explained by corresponding similarity and consistency assessment scores. The use of the TSECA framework enabled us to identify some between-trial differences in participants, interventions, and outcome measures, although we were uncertain about the impact of the most-observed between-trial differences. After further detailed consideration, we concluded that the indirect comparison may be invalid because of different baseline risks (as reflected by the event rate in the placebo arms) between the two sets of trials in the indirect comparison. The difference in the baseline risk may be caused by an imbalance in the distribution of certain prognostic variables across trials. The indirect and mixed treatment comparisons will be invalid if any of these prognostic variables are also known or unknown modifiers of the relative treatment effect [12]. Therefore, the case study suggested that the comprehensive assessment of trial similarity and evidence consistency can provide important evidence on the validity of the indirect and mixed treatment comparisons.

## 5 Conclusions

The assessment of trial similarity and evidence consistency for indirect or mixed treatment comparisons seeks to answer two key questions. The first question is whether there are considerable differences in participants, interventions, outcome measures, and the risk of bias between different sets of trials. The second question is whether the relative treatment effect may have been modified by any of the observed between-trial differences. The TSECA framework evaluated in this study may be helpful for answering the first question. Although any overall trial similarity or evidence consistency scores are unlikely to be accurate enough for predicting the inconsistency between direct and indirect estimates, we can use this framework to systematically and explicitly collate relevant evidence for identifying between-trial differences that may threaten the validity of indirect or mixed treatment comparisons.

## Figures and Tables

**Table 1:** Similarity/consistency scores and statistically significant inconsistency between direct and indirect estimates

Trial similarity scores and other variables (grouped by quantile points)	*N* (%) with significant inconsistency	*P*-value for difference between subgroups
Average trial clinical similarity score		
≤3.51	4/25 (16.0)	0.114
>3.51 & ≤3.84	2/22 (9.1)	
>3.84 & ≤4.12	8/25 (32.0)	
>4.12	2/22 (9.1)	
Trial quality similarity score		
≤3.67	3/23 (13.0)	0.891
>3.67 & ≤4.235	4/24 (16.7)	
>4.235 & ≤4.62	4/24 (16.7)	
>4.62	5/23 (21.7)	
Evidence consistency scores		
≤3.36	3/24 (12.5)	0.831
>3.36 & ≤3.645	4/23 (17.4)	
<3.645 & ≤3.90	5/22 (22.7)	
>3.90	4/25 (16.0)	
Minimal similarity or consistency scores		
≤1.75	6/31 (19.4)	0.734
>1.75 & ≤2.00	5/32 (15.6)	
>2.00 & ≤2.25	5/26 (19.2)	
>2.25	0/5 (0.0)	
