# Supplementary material for: Overall similarity and consistency assessment scores are not sufficiently accurate for predicting discrepancy between direct and indirect comparison estimates
Source: J Clin Epidemiol. 2013 Feb;66(2):184–91. doi: 10.1016/j.jclinepi.2012.06.022 (PMC3537077; doi:10.1016/j.jclinepi.2012.06.022)
Supplement: Tables S1–S9 and Figs. S1 and S2 [file mmc1.pdf]

Assessment of trial similarity and evidence consistency for indirect treatment comparisons: an empirical investigation

LIST OF SUPPLEMENTAL FILES

Supporting Figures:

Figure S1: Agreement between two independent assessors in the assessment of trial similarity and evidence consistency

Figure S2: Mean disagreement (95% CI) between two independent assessors in final similarity and consistency scores.

Supporting Tables:

Table S1: Main characteristics of included CSRs and similarity/consistency assessment scores

Table S2: Evidence table for data extraction from trials included in CSRs

Table S3: TSA sheet for clinical similarity assessment

Table S4: QSA sheet for Quality assessment of trials in meta-analysis

Table S5: ECA sheet for evidence consistency assessment

Table S6: Evidence table – topical azelaic acid vs. topical metronidazole for rosacea (case study CD003262)

Table S7: Clinical similarity assessment results - case study (CD003262)

Table S8: ECA- evidence consistency assessment results –case study (CD003262)

Table S9: Assessment of quality of studies included – case study (CD003262)

Figure S1: Agreement between two independent assessors in the assessment of trial similarity and evidence consistency (Note: d –difference in the score between two assessors, SD –standard deviation, SE – standard error)

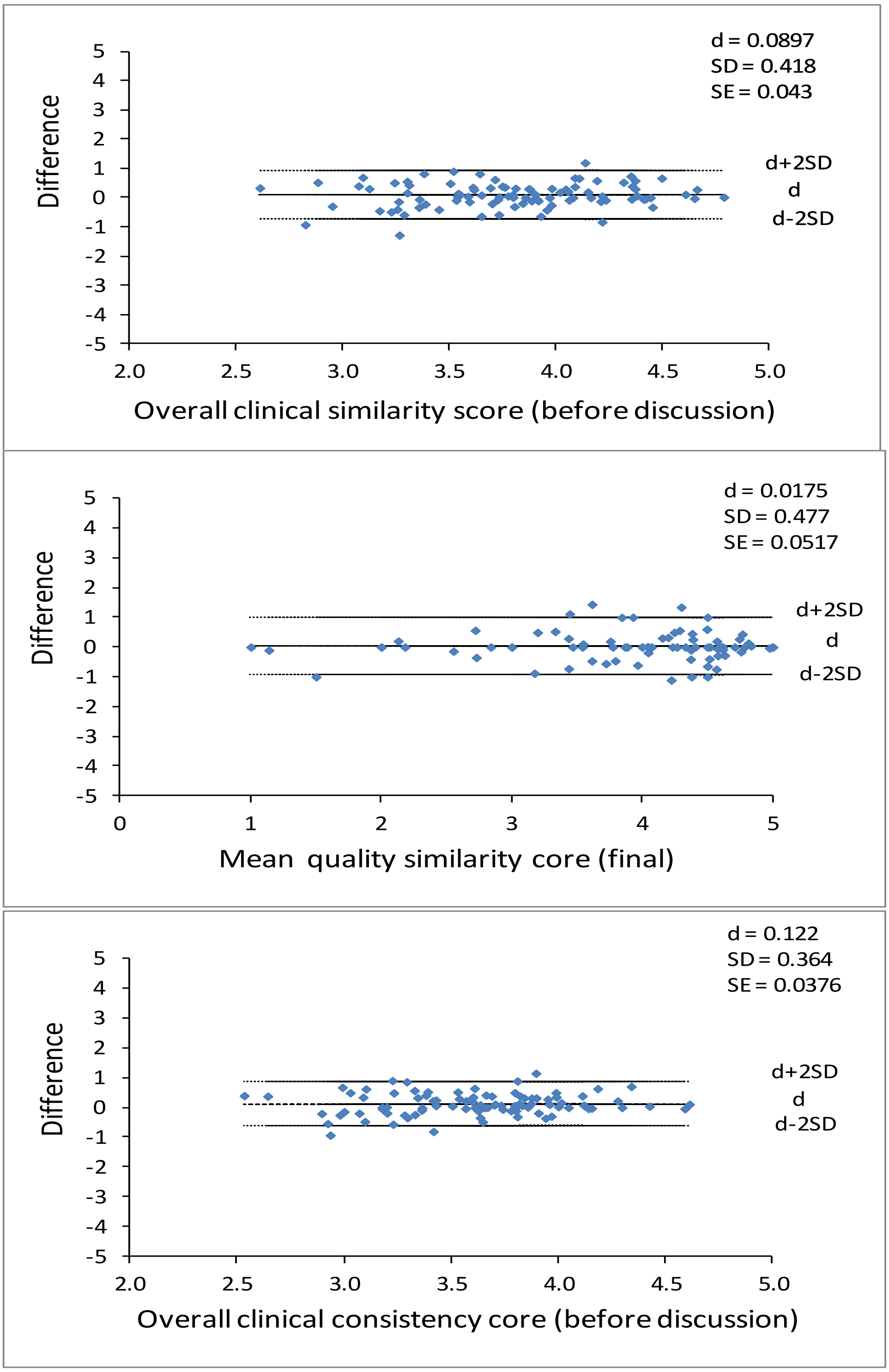

Figure S2: Mean disagreement (95% CI) between two independent assessors in final similarity and consistency scores (d=0 refers no difference in similarity/consistency scores between the two assessors)

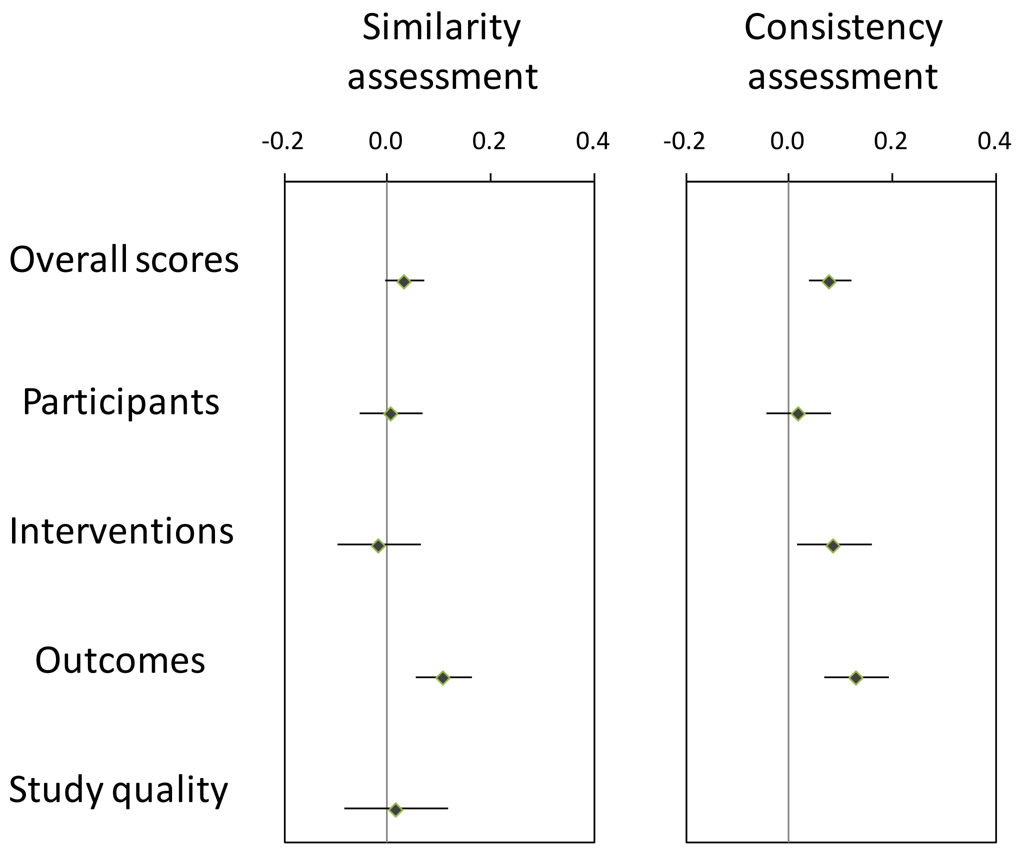

Table S1: Main characteristics of included CSRs and similarity/consistency assessment scores (TSA – Trial Similarity Assessment; ECA – Evidence Consistency Assessment; PSS – participant similarity score; ISS –intervention similarity score, OSS – outcome similarity score, TSS – average total similarity score, QSS –quality similarity score; PCS –participant consistency score, ICS –intervention consistency score, OCS – outcome consistency score, TCS –average total consistency score)

CSR	Clinical indications	Treatment (B; C; A)	Outcome measures	No.of trials (patients): BvC, AvB, AvC	Delta (SE)	TSA scores		ECA scores	
**CD000053**	Urinary Schistosomiasis	B: Praziquantel<br>C: Metrifonate<br>A: Placebo	Parasitological failure (1-12mon)	5 (1757)<br>2 (473)<br>4 (533)	-1.005<br>(1.442)	PSS<br>ISS<br>OSS<br>TSS<br>QSS	3.42<br>3.96<br>3.25<br>3.54<br>2.84	PCS<br>ICS<br>OCS<br>TCS	3.09<br>4.11<br>3.17<br>3.45
**CD000146**	Smokers	B: NRT patch<br>C: NRT spray<br>A: Placebo or no-NRT	Cessation rate	2 (1272)<br>41 (18237)<br>4 (887)	-0.348<br>(0.273)	PSS<br>ISS<br>OSS<br>TSS<br>QSS	3.00<br>2.44<br>3.17<br>2.87<br>4.92	PCS<br>ICS<br>OCS<br>TCS	2.92<br>2.38<br>3.17<br>2.82
**CD000165**	Smokers	B: Multiple visit<br>C: single visit<br>A: Usual care	Smoking cessation	5 (1254)<br>5 (4174)<br>18 (14675)	-0.003<br>(0.271)	PSS<br>ISS<br>OSS<br>TSS<br>QSS	3.05<br>2.75<br>3.17<br>2.99<br>3.75	PCS<br>ICS<br>OCS<br>TCS	3.00<br>2.68<br>3.17<br>2.95
**CD000184**	Breech presentation	B: Betamimentic<br>C: Nitric oxide donor<br>A: Control	Failed external cephalic version	2 (109)<br>6 (617)<br>2 (156)	-0.131<br>(0.940)	PSS<br>ISS<br>OSS<br>TSS<br>QSS	4.05<br>4.67<br>5.00<br>4.57<br>3.95	PCS<br>ICS<br>OCS<br>TCS	3.55<br>3.43<br>5.00<br>3.99
**CD000195**	Asthma (acute exacerbation)	B: Intramuscular corticosteroids<br>C: Oral corticosteroids;<br>A: Placebo	Relapse rates	1 (36)<br>1 (56)<br>3 (221)	2.130<br>(3.29)	PSS<br>ISS<br>OSS<br>TSS<br>QSS	3.29<br>3.25<br>4.00<br>3.51<br>4.50	PCS<br>ICS<br>OCS<br>TCS	3.00<br>3.18<br>4.00<br>3.39

CSR	Clinical indications	Treatment (B; C; A)	Outcome measures	No.of trials (patients): BvC, AvB, AvC	Delta (SE)	TSA scores		ECA scores	
**CD000218**	Trichomoniasis	B: Metronidazole<br>C: Nimorazole<br>A: Ornidazole	No parasitological cure	7 (920)<br>1 (48)<br>1 (58)	-3.179<br>(1.831)	PSS<br>ISS<br>OSS<br>TSS<br>QSS	3.38<br>3.42<br>3.25<br>3.35<br>3.63	PCS<br>ICS<br>OCS<br>TCS	3.02<br>3.18<br>3.25<br>3.15
**CD000227**	Fractures associated with involution or osteoporosis	B: Vitamins D + Calcium<br>C: Vitamin D<br>A: Placebo or control	New hip fracture	3 (2832)<br>4 (15848)<br>4 (5283)	0.533<br>(0.257)	PSS<br>ISS<br>OSS<br>TSS<br>QSS	3.07<br>2.59<br>4.34<br>3.33<br>4.79	PCS<br>ICS<br>OCS<br>TCS	3.07<br>3.04<br>4.34<br>3.48
**CD000256**	Uncomplicated malaria	B: Artesunate<br>C: Artemether<br>A: Chlororquine	Parasite clearance at day 7	4 (661)<br>1 (103)<br>1 (106)	1.765<br>(1.058)	PSS<br>ISS<br>OSS<br>TSS<br>QSS	3.42<br>5.00<br>5.00<br>4.47<br>2.00	PCS<br>ICS<br>OCS<br>TCS	2.88<br>4.14<br>5.00<br>4.01
**CD000305**	Deep vein thrombosis	B: LMW heparin<br>C: Unfractioned heparin<br>A: Placebo or no heparin	Any DVT	3 (247)<br>3 (177)<br>10 (816)	-0.244<br>(0.720)	PSS<br>ISS<br>OSS<br>TSS<br>QSS	3.85<br>5.00<br>3.17<br>4.01<br>4.31	PCS<br>ICS<br>OCS<br>TCS	3.80<br>3.82<br>3.17<br>3.60
**CD000307**	Schizophrenia	B: Fluphenazine decanoate<br>C: Fluphenazine enanthate<br>A: Oral neuroleptic	Movement disorders general	2 (49)<br>4 (303)<br>1 (31)	-2.464<br>(1.189)	PSS<br>ISS<br>OSS<br>TSS<br>QSS	2.36<br>3.17<br>2.50<br>2.67<br>3.89	PCS<br>ICS<br>OCS<br>TCS	2.36<br>3.54<br>2.50<br>2.80
**CD000320**	Scabies	B: Permethrin<br>C: Lindane<br>A: Ivermectin	Treatment failure in clinically diagnosed cases	5 (753)<br>1 (85)<br>2 (193)	2.640<br>(1.122)	PSS<br>ISS<br>OSS<br>TSS<br>QSS	3.38<br>4.80<br>4.09<br>4.09<br>3.67	PCS<br>ICS<br>OCS<br>TCS	3.46<br>4.61<br>4.09<br>4.05

CSR	Clinical indications	Treatment (B; C; A)	Outcome measures	No.of trials (patients): BvC, AvB, AvC	Delta (SE)	TSA scores		ECA scores	
**CD000402**	Post-menopausal women	B: Oestrogen + Progestragen (sequential)<br>C: Oestrogen<br>A: Placebo	Endometrial hyperplasia at 12 months	4 (2117)<br>1 (49)<br>3 (1699)	-1.229<br>(1.374)	PSS<br>ISS<br>OSS<br>TSS<br>QSS	3.63<br>4.84<br>5.00<br>4.49<br>2.62	PCS<br>ICS<br>OCS<br>TCS	3.63<br>3.75<br>5.00<br>4.13
**CD000527**	Severe malaria	B: Arestunate<br>C: Artesmisinin<br>A: Quinine	Mortality	3 (253)<br>2 (231)<br>1 (65)	0.440<br>(1.015)	PSS<br>ISS<br>OSS<br>TSS<br>QSS	2.46<br>5.00<br>4.00<br>3.82<br>4.82	PCS<br>ICS<br>OCS<br>TCS	2.46<br>4.21<br>4.00<br>3.56
**CD000978**	Oral mucositis	B: GM-CSF<br>C: Scuralfate<br>A: Control	Mucositis (0-2 versus 3+)	1 (40)<br>6 (358)<br>6 (423)	-0.497<br>(0.542)	PSS<br>ISS<br>OSS<br>TSS<br>QSS	3.32<br>3.67<br>3.13<br>3.37<br>4.55	PCS<br>ICS<br>OCS<br>TCS	3.29<br>2.95<br>3.13<br>3.12
**CD001103**	Venous leg ulcer	B: Low adherent<br>C: Hydrocolloid dressing<br>A: Foam dressing	Total number of ulcers healed	8 (792)<br>2 (203)<br>4 (311)	0.194<br>(0.44)	PSS<br>ISS<br>OSS<br>TSS<br>QSS	3.57<br>4.15<br>4.09<br>3.93<br>4.29	PCS<br>ICS<br>OCS<br>TCS	3.53<br>4.01<br>4.09<br>3.87
**CD001136**	Caesarean section	B: 2nd/3rd generation cephalosporin<br>C: 1st generation cephalosporin<br>A: Ampicillin	Endometritis	9 (2693)<br>1 (119)<br>1 (100)	0.694<br>(1.313)	PSS<br>ISS<br>OSS<br>TSS<br>QSS	3.40<br>3.17<br>3.25<br>3.27<br>3.00	PCS<br>ICS<br>OCS<br>TCS	3.40<br>3.58<br>3.25<br>3.41
**CD001169**	Influenza A	B: Rimanradine<br>C: Amantadine<br>A: Placeco	Influenza cases	2 (455)<br>1 (222)<br>9 (4194)	0.720<br>(1.03)	PSS<br>ISS<br>OSS<br>TSS<br>QSS	2.94<br>4.23<br>3.50<br>3.56<br>2.90	PCS<br>ICS<br>OCS<br>TCS	2.94<br>3.88<br>3.50<br>3.44

CSR	Clinical indications	Treatment (B; C; A)	Outcome measures	No.of trials (patients): BvC, AvB, AvC	Delta (SE)	TSA scores		ECA scores	
**CD001209**	Urinary tract infection	B: Norflozacin<br>C: Nitrofurantoin<br>A: Placebo	Microbiological recurrence	1 (52)<br>2 (59)<br>2 (69)	1.936<br>(1.895)	PSS<br>ISS<br>OSS<br>TSS<br>QSS	4.32<br>4.96<br>4.00<br>4.43<br>4.59	PCS<br>ICS<br>OCS<br>TCS	3.67<br>3.83<br>3.92<br>3.80
**CD001217**	thromboprophylaxis in colorectal surgery	B: LMW Heparin coverage<br>C: LD Heparin<br>A: Placebo or no treatment	DVT	3 (1177)<br>1 (11)<br>3 (168)	0.904<br>(0.838)	PSS<br>ISS<br>OSS<br>TSS<br>QSS	4.38<br>5.00<br>3.33<br>4.24<br>3.00	PCS<br>ICS<br>OCS<br>TCS	3.64<br>4.33<br>3.33<br>3.76
**CD001319**	Fluid resustication	B: Albumin/PPF<br>C: HES<br>A: Gelatine	Death	13 (819)<br>1 (475)<br>11 (1024)	-0.209<br>(0.34)	PSS<br>ISS<br>OSS<br>TSS<br>QSS	2.75<br>3.13<br>3.79<br>3.22<br>4.37	PCS<br>ICS<br>OCS<br>TCS	2.75<br>2.97<br>3.79<br>3.17
**CD001324**	Unprotected intercourse	B: Mifepristone (25-50 mg)<br>C: Mifepestone <25mg<br>A: Levonorgestrel	pregnancy	18 (11242)<br>15 (3743)<br>8 (7916)	-0.090<br>(0.324)	PSS<br>ISS<br>OSS<br>TSS<br>QSS	3.59<br>5.00<br>4.17<br>4.25<br>1.19	PCS<br>ICS<br>OCS<br>TCS	3.46<br>4.93<br>4.17<br>4.19
**CD001434**	Tinea pedis	B: Allylamines<br>C: Azoles<br>A: Placebo	short-term (2 weeks) treatment failure	10 (1519)<br>9 (928)<br>5 (329)	-0.475<br>(0.557)	PSS<br>ISS<br>OSS<br>TSS<br>QSS	2.92<br>4.96<br>4.04<br>3.97<br>4.61	PCS<br>ICS<br>OCS<br>TCS	2.90<br>4.21<br>4.09<br>3.73
**CD001449**	Hypertension during pregnancy	B: Calcium channel blockers<br>C: Hyrazaline<br>A: Labetolol	Persistent high BP	3 (199)<br>1 (60)<br>1 (20)	-2.487<br>(1.164)	PSS<br>ISS<br>OSS<br>TSS<br>QSS	3.53<br>3.08<br>3.33<br>3.31<br>3.47	PCS<br>ICS<br>OCS<br>TCS	3.23<br>3.71<br>3.33<br>3.42

CSR	Clinical indications	Treatment (B; C; A)	Outcome measures	No.of trials (patients): BvC, AvB, AvC	Delta (SE)	TSA scores		ECA scores	
**CD001501**	Menorrhagia	B: Laser ablation<br>C: TCRE<br>A: Balloon ablation	Participant satisfaction at 12 mon	1 (321)<br>1 (57)<br>1 (75)	-1.176<br>(1.397)	PSS<br>ISS<br>OSS<br>TSS<br>QSS	4.17<br>5.00<br>4.17<br>4.45<br>4.00	PCS<br>ICS<br>OCS<br>TCS	4.11<br>5.00<br>4.17<br>4.43
**CD001535**	lower urinary tract infections	B: Long course<br>C: Short course<br>A: Single	Persistent UIT	3 (431)<br>3 (453)<br>5 (356)	-0.686<br>(0.884)	PSS<br>ISS<br>OSS<br>TSS<br>QSS	2.88<br>4.00<br>4.09<br>3.65<br>4.92	PCS<br>ICS<br>OCS<br>TCS	2.88<br>3.33<br>4.09<br>3.43
**CD001751**	Dysmenorrhea	B: Diclofenac<br>C: Nimesulide<br>A: Placebo	Subjective pain relief	1 (304)<br>2 (80)<br>1 (37)	-2.349<br>(1.019)	PSS<br>ISS<br>OSS<br>TSS<br>QSS	3.96<br>4.92<br>2.34<br>3.74<br>4.50	PCS<br>ICS<br>OCS<br>TCS	3.52<br>4.54<br>2.34<br>3.46
**CD001781**	Cutaneous warts	B: Cryotherapy<br>C: Salicylic + lactic acid<br>A: Placebo/no treatment	Cure rate	3 (320)<br>2 (69)<br>5 (322)	-1.469<br>(0.767)	PSS<br>ISS<br>OSS<br>TSS<br>QSS	3.33<br>4.21<br>4.04<br>3.86<br>3.77	PCS<br>ICS<br>OCS<br>TCS	3.33<br>3.50<br>4.00<br>3.61
**CD001782**	Photodamage skin	B: Topical tretinoin 0.05%<br>C: Topical tretinoin 0.01%<br>A: Placebo	Face overall improved - investigator’s assessment	2 (318)<br>1 (116)<br>1 (34)	-2.323<br>(0.971)	PSS<br>ISS<br>OSS<br>TSS<br>QSS	3.19<br>5.00<br>3.54<br>3.91<br>4.00	PCS<br>ICS<br>OCS<br>TCS	3.14<br>5.00<br>3.62<br>3.92
**CD001886**	Cardiac surgery	B: Tranexamic acid<br>C: Aprotinin<br>A: Control	Mortality (cardia surgery subgroup)	5 (1401)<br>9 (1080)<br>28 (5820)	-0.228<br>(0.582)	PSS<br>ISS<br>OSS<br>TSS<br>QSS	4.13<br>3.79<br>3.42<br>3.78<br>3.47	PCS<br>ICS<br>OCS<br>TCS	3.63<br>3.75<br>3.25<br>3.54

CSR	Clinical indications	Treatment (B; C; A)	Outcome measures	No.of trials (patients): BvC, AvB, AvC	Delta (SE)	TSA scores		ECA scores	
**CD001896**	Dysmenorrhoea	B: Laparoscopic presacral neurectomy<br>C: Laparoscopic unterine nerve ablation (LUNA)<br>A: Control	Pain relief up to 12 months	1 (68)<br>1 (126)<br>2 (68)	-2.367<br>(0.947)	PSS<br>ISS<br>OSS<br>TSS<br>QSS	3.54<br>4.38<br>4.13<br>4.02<br>4.62	PCS<br>ICS<br>OCS<br>TCS	3.34<br>4.05<br>4.13<br>3.84
**CD001951**	Schizophrenia	B: Haloperidol 15-35mg/d<br>C: Haloperidol 7.5-15mg/d<br>A: Haloperidol >35mg/d	Leaving study early	5 (208)<br>1 (128)<br>1 (48)	1.854<br>(1.613)	PSS<br>ISS<br>OSS<br>TSS<br>QSS	3.36<br>4.04<br>3.13<br>3.51<br>4.00	PCS<br>ICS<br>OCS<br>TCS	3.15<br>3.65<br>3.13<br>3.31
**CD001955**	Croup	B: Budesonide<br>C: Dexamethasone<br>A: Placebo	Croup scores 6 hours NB; Westley score	4 (326)<br>3 (173)<br>2 (76)	2.909<br>(1.581)	PSS<br>ISS<br>OSS<br>TSS<br>QSS	3.58<br>5.00<br>4.83<br>4.47<br>4.33	PCS<br>ICS<br>OCS<br>TCS	3.36<br>3.59<br>4.75<br>3.90
**CD001960**	dyspepsia	B: PPI<br>C: H2RA<br>A: Antacids	Global symptom response	2 (739)<br>9 (2749)<br>11 (1787)	-0.507<br>(0.324)	PSS<br>ISS<br>OSS<br>TSS<br>QSS	2.94<br>5.00<br>3.25<br>3.73<br>3.85	PCS<br>ICS<br>OCS<br>TCS	2.94<br>3.90<br>3.25<br>3.36
**CD001961**	Dyspepsia	B: PPI<br>C: H2RA<br>A: Antacids	Global assessment	3 (1267)<br>3 (1615)<br>1 (255)	-0.002<br>(0.364)	PSS<br>ISS<br>OSS<br>TSS<br>QSS	2.27<br>3.44<br>1.96<br>2.56<br>1.00	PCS<br>ICS<br>OCS<br>TCS	2.27<br>3.20<br>1.96<br>2.48
**CD002060**	Neonatal jaundice	B: Biliblanket<br>C: Wallaby<br>A: Conventional Phototherapy	Change in serum bilirubin concentration over total period (% change /hr)	1 (60)<br>8 (513)<br>3 (164)	0.261<br>(0.275)	PSS<br>ISS<br>OSS<br>TSS<br>QSS	3.60<br>3.34<br>4.17<br>3.70<br>4.82	PCS<br>ICS<br>OCS<br>TCS	2.85<br>3.32<br>4.17<br>3.45

CSR	Clinical indications	Treatment (B; C; A)	Outcome measures	No.of trials (patients): BvC, AvB, AvC	Delta (SE)	TSA scores		ECA scores	
**CD002095**	Reflux dis -positive endoscopy	B: PPI<br>C: H2RA<br>A: Antacids	Heartburn remission	7 (3147)<br>2 (760)<br>2 (1013)	0.570<br>(0.27)	PSS<br>ISS<br>OSS<br>TSS<br>QSS	3.69<br>5.00<br>3.17<br>3.95<br>4.90	PCS<br>ICS<br>OCS<br>TCS	3.53<br>4.54<br>3.17<br>3.74
**CD002123**	dysmenorrhoea	B: High frequency TENS<br>C: Low frequency TENS<br>A: Placebo TENS	Pain relief – overall experience	1 (42)<br>1 (64)<br>1 (8)	2.275<br>(2.103)	PSS<br>ISS<br>OSS<br>TSS<br>QSS	3.71<br>3.83<br>4.17<br>3.90<br>2.00	PCS<br>ICS<br>OCS<br>TCS	3.71<br>3.97<br>3.33<br>3.67
**CD002251**	Hypotension during caesarean section	B: Ephedrine<br>C: Crystalloid<br>A: Control	Women with hypotension requiring intervention	4 (293)<br>6 (350)<br>1 (140)	-0.103<br>(0.692)	PSS<br>ISS<br>OSS<br>TSS<br>QSS	3.69<br>3.75<br>3.33<br>3.59<br>4.52	PCS<br>ICS<br>OCS<br>TCS	3.69<br>3.83<br>3.33<br>3.62
**CD002252**	Hypertension during pregnancy	B: Beta-blocker<br>C: Methyldopa<br>A: Control (none)	Proteinuria/pre-eclampsia	9 (804)<br>8 (883)<br>2 (267)	0.483<br>(0.558)	PSS<br>ISS<br>OSS<br>TSS<br>QSS	3.30<br>3.84<br>3.00<br>3.38<br>3.72	PCS<br>ICS<br>OCS<br>TCS	3.30<br>3.23<br>3.00<br>3.18
**CD002296**	gastroduodenal ulcers	B: Proton-pump inhibitor<br>C: H2-receptor antagonists (H2RA)<br>A: Placebo	Total endoscopic ulcers	1 (425)<br>5 (1216)<br>5 (1186)	-0.715<br>(0.406)	PSS<br>ISS<br>OSS<br>TSS<br>QSS	3.69<br>4.67<br>4.00<br>4.12<br>4.33	PCS<br>ICS<br>OCS<br>TCS	3.67<br>4.04<br>4.00<br>3.90
**CD002780**	Dental Caries	B: Fluoride toothpast<br>C: Fluoride mouthrinse<br>A: Fluoride varnish	Leaving study early	5 (2752)<br>1 (193)<br>2 (626)	-0.180<br>(0.674)	PSS<br>ISS<br>OSS<br>TSS<br>QSS	3.40<br>4.54<br>3.63<br>3.86<br>5.00	PCS<br>ICS<br>OCS<br>TCS	3.40<br>3.93<br>3.63<br>3.65

CSR	Clinical indications	Treatment (B; C; A)	Outcome measures	No.of trials (patients): BvC, AvB, AvC	Delta (SE)	TSA scores		ECA scores	
**CD002898**	herpes simplex virus epithelial keratitis	B: Acyclovir<br>C: Idoxuridine<br>A: Trifluridine	Healing at 14 days	11 (600)<br>3 (140)<br>4 (219)	-0.063<br>(0.80)	PSS<br>ISS<br>OSS<br>TSS<br>QSS	3.71<br>4.17<br>3.33<br>3.74<br>5.00	PCS<br>ICS<br>OCS<br>TCS	3.29<br>3.90<br>3.33<br>3.51
**CD002916**	Malignant pleural effusion	B: Talc<br>C: Tetracycline<br>A: Control	No recurrence of effusions	3 (103)<br>2 (82)<br>2 (51)	-2.490<br>(2.678)	PSS<br>ISS<br>OSS<br>TSS<br>QSS	2.92<br>4.21<br>3.33<br>3.49<br>4.23	PCS<br>ICS<br>OCS<br>TCS	2.92<br>3.36<br>3.33<br>3.20
**CD003101**	Induction of labour	B: Prostaglandin E2<br>C: Prostaglandin F2a (PGF2a)<br>A: Placebo/no treatment	Caesarean section	2 (107)<br>31 (6211)<br>2 (355)	-0.400<br>(0.634)	PSS<br>ISS<br>OSS<br>TSS<br>QSS	3.63<br>3.27<br>4.17<br>3.69<br>4.50	PCS<br>ICS<br>OCS<br>TCS	3.63<br>3.65<br>4.17<br>3.81
**CD003167**	glaucoma and ocular hypertension	B: Betaxolol<br>C: Timolol<br>A: Placebo	Drop-out due to drug-related adverse events	3 (195)<br>1 (356)<br>2 (127)	-0.023<br>(0.939)	PSS<br>ISS<br>OSS<br>TSS<br>QSS	3.81<br>4.96<br>4.04<br>4.27<br>4.71	PCS<br>ICS<br>OCS<br>TCS	3.71<br>4.61<br>4.04<br>4.12
**CD003187**	Hodgkin's disease-early stage	B: Radiotherapy<br>C: Chemotherapy<br>A: Combined chemo-radiotherapy	Overall survival	2 (299)<br>11 (2744)<br>3 (495)	0.124<br>(0.417)	PSS<br>ISS<br>OSS<br>TSS<br>QSS	2.67<br>3.36<br>3.92<br>3.31<br>3.68	PCS<br>ICS<br>OCS<br>TCS	2.63<br>3.07<br>4.00<br>3.23
**CD003209**	Distal radial fractures	B: Percuctaneous pinning;<br>C: External fixation<br>A: Plaster cast	Functional grading - not excellent	2 (99)<br>4 (233)<br>8 (489)	-0.902<br>(0.986)	PSS<br>ISS<br>OSS<br>TSS<br>QSS	4.03<br>5.00<br>3.17<br>4.07<br>4.97	PCS<br>ICS<br>OCS<br>TCS	3.53<br>4.71<br>3.17<br>3.80

CSR	Clinical indications	Treatment (B; C; A)	Outcome measures	No.of trials (patients): BvC, AvB, AvC	Delta (SE)	TSA scores		ECA scores	
**CD003226**	migraine	B: Topiramate 200mg<br>C: Topiramate 100mg<br>A: Placebo	50% or greater reduction in migraine frequency	3 (756)<br>1 (72)<br>2 (70)	0.154<br>(0.838)	PSS<br>ISS<br>OSS<br>TSS<br>QSS	3.38<br>5.00<br>2.59<br>3.65<br>3.43	PCS<br>ICS<br>OCS<br>TCS	3.13<br>4.64<br>2.59<br>3.45
**CD003245**	reflux disease	B: PPI<br>C: H2 RA<br>A: Placebo	Relapse (oesophagitis)	10 (1583)<br>9 (1385)<br>4 (655)	0.143<br>(0.517)	PSS<br>ISS<br>OSS<br>TSS<br>QSS	3.67<br>4.84<br>4.50<br>4.33<br>4.99	PCS<br>ICS<br>OCS<br>TCS	3.61<br>4.36<br>4.05<br>4.00
**CD003261**	Impetigo	B: Mupirocin<br>C: Fusidic acid<br>A: Placebo	Cure/improvement	4 (440)<br>3 (173)<br>1 (156)	-0.687<br>(0.592)	PSS<br>ISS<br>OSS<br>TSS<br>QSS	2.17<br>3.96<br>3.21<br>3.11<br>2.23	PCS<br>ICS<br>OCS<br>TCS	2.11<br>3.52<br>3.21<br>2.94
**CD003262**	rosacea	B: Topical azelaic acid<br>C: Topical metronidazole<br>A; Placebo	Physician’s global evaluation of improvement	1 (251)<br>3 (778)<br>3 (313)	-1.590<br>(0.449)	PSS<br>ISS<br>OSS<br>TSS<br>QSS	3.67<br>4.71<br>3.17<br>3.85<br>4.41	PCS<br>ICS<br>OCS<br>TCS	3.67<br>4.05<br>3.17<br>3.63
**CD003352**	cocaine dependence	B: Amantadine<br>C: Bromocriptine<br>A: Placebo	Positive urine sample for cocaine metabolites	1 (14)<br>1 (30)<br>1 (29)	0.010<br>(1.696)	PSS<br>ISS<br>OSS<br>TSS<br>QSS	3.25<br>4.92<br>4.09<br>4.08<br>5.00	PCS<br>ICS<br>OCS<br>TCS	2.96<br>4.22<br>4.09<br>3.75
**CD003370**	Advanced breast cancer	B: Aminoglutethimide<br>C: Letrozole<br>A: non-Al	No clinical benefit (assessable)	1 (335)<br>4 (1637)<br>5 (637)	-0.434<br>(0.309)	PSS<br>ISS<br>OSS<br>TSS<br>QSS	3.13<br>3.38<br>3.67<br>3.39<br>3.08	PCS<br>ICS<br>OCS<br>TCS	3.17<br>3.20<br>3.67<br>3.34

CSR	Clinical indications	Treatment (B; C; A)	Outcome measures	No.of trials (patients): BvC, AvB, AvC	Delta (SE)	TSA scores		ECA scores	
**CD003385**	Bulimia Nervosa	B: Antidepressant + Csychotherapy<br>A: Antidepressent<br>C: Psychotherapy	Remission	4 (141)<br>2 (107)<br>1 (88)	-1.156<br>(0.747)	PSS<br>ISS<br>OSS<br>TSS<br>QSS	3.59<br>1.29<br>3.25<br>2.71<br>4.88	PCS<br>ICS<br>OCS<br>TCS	3.25<br>1.35<br>3.25<br>2.62
**CD003388**	Post traumatic stress disorder	B: EMDR<br>C: TFCBT<br>A: Waiting list/usual care	PTSD diagnosis after treatment	7 (260)<br>3 (72)<br>12 (620)	0.198<br>(1.38)	PSS<br>ISS<br>OSS<br>TSS<br>QSS	2.90<br>2.84<br>3.06<br>2.93<br>4.37	PCS<br>ICS<br>OCS<br>TCS	2.82<br>2.66<br>3.06<br>2.85
**CD003431**	Anal fissure	B: Botox<br>C: Nitroglycerin ointment (TN)<br>A: Placebo	Non-healing	4 (187)<br>3 (136)<br>15 (1190)	-0.503<br>(1.363)	PSS<br>ISS<br>OSS<br>TSS<br>QSS	3.08<br>4.46<br>3.92<br>3.82<br>3.68	PCS<br>ICS<br>OCS<br>TCS	2.83<br>3.50<br>3.92<br>3.42
**CD003534**	Chronic asthma	B: FP 400-500 mcg/d<br>C: FP 100 mcg/d<br>A: FP 200 mcg/d	Change in FEV1 (L)	4 (511)<br>8 (1226)<br>3 (404)	-0.222<br>(0.147)	PSS<br>ISS<br>OSS<br>TSS<br>QSS	3.34<br>3.52<br>3.80<br>3.55<br>4.63	PCS<br>ICS<br>OCS<br>TCS	3.34<br>3.04<br>3.80<br>3.39
**CD003584**	Tinea pedis	B: Terbinafine<br>C: Itraconazole<br>A: Placebo	Cure (week 8)	3 (295)<br>1 (41)<br>1 (48)	0.446<br>(1.842)	PSS<br>ISS<br>OSS<br>TSS<br>QSS	4.13<br>4.83<br>5.00<br>4.65<br>4.00	PCS<br>ICS<br>OCS<br>TCS	3.34<br>3.97<br>5.00<br>4.10
**CD003592**	anxiety disorder	B: Paroxetine<br>C: Imipramine<br>A: Placebo	No treatment response	1 (56)<br>1 (324)<br>1 (113)	-0.980<br>(0.987)	PSS<br>ISS<br>OSS<br>TSS<br>QSS	3.82<br>4.86<br>5.00<br>4.56<br>4.00	PCS<br>ICS<br>OCS<br>TCS	3.77<br>4.38<br>5.00<br>4.38

CSR	Clinical indications	Treatment (B; C; A)	Outcome measures	No.of trials (patients): BvC, AvB, AvC	Delta (SE)	TSA scores		ECA scores	
**CD003664**	allergy and food intolerance in infants	B: Extensively hydrolysed formula<br>C: Cow's milk formula<br>A: Partially hydrolysed formula	Any allergy	2 (1561)<br>1 (246)<br>5 (425)	0.726<br>(0.387)	PSS<br>ISS<br>OSS<br>TSS<br>QSS	3.44<br>4.35<br>3.92<br>3.90<br>3.29	PCS<br>ICS<br>OCS<br>TCS	3.44<br>4.17<br>3.92<br>3.84
**CD003677**	hysterectomy	B: Laparoscopic hysterectomy (LH)<br>C: Abdominal hysterectomy (AH)<br>A: Vaginal hysterectomy (VH)	Length of hospital stay (days)	9 (948)<br>2 (157)<br>2 (155)	-1.846<br>(0.603)	PSS<br>ISS<br>OSS<br>TSS<br>QSS	4.45<br>4.13<br>4.09<br>4.22<br>4.07	PCS<br>ICS<br>OCS<br>TCS	4.43<br>3.86<br>4.09<br>4.12
**CD003723**	status epilepticus	B: Lorazepam<br>C: Diazepam<br>A: Midazolam	Non-cessation of seizures	3 (264)<br>1 (27)<br>1 (40)	-2.644<br>(1.399)	PSS<br>ISS<br>OSS<br>TSS<br>QSS	2.73<br>3.63<br>4.09<br>3.48<br>3.00	PCS<br>ICS<br>OCS<br>TCS	2.61<br>2.97<br>4.09<br>3.22
**CD003738**	cataract	B: PMMA (polymethyl methacrylate)<br>C: Silicone<br>A: Acrylic	YAG rate	4 (244)<br>2 (229)<br>5 (334)	-1.947<br>(1.046)	PSS<br>ISS<br>OSS<br>TSS<br>QSS	4.50<br>4.26<br>4.25<br>4.34<br>4.10	PCS<br>ICS<br>OCS<br>TCS	4.50<br>4.10<br>3.75<br>4.12
**CD003774**	solid organ transplant recipients	B: Ganiciclovir<br>C: Acicolvir<br>A: Placebo	CMV organ involvement	7 (1034)<br>7 (769)<br>3 (216)	-0.866<br>(0.674)	PSS<br>ISS<br>OSS<br>TSS<br>QSS	4.19<br>3.42<br>3.75<br>3.79<br>4.88	PCS<br>ICS<br>OCS<br>TCS	4.13<br>3.41<br>3.75<br>3.76
**CD003807**	oral candidiasis in cancer patients	B: Drugs absorbed from GI tract<br>C: Drugs not absorbed from GI<br>A: Placebo or no treatment.	Oral candidiasis present	8 (2103)<br>6 (1123)<br>7 (362)	-0.992<br>(0.642)	PSS<br>ISS<br>OSS<br>TSS<br>QSS	3.27<br>3.67<br>3.25<br>3.40<br>3.78	PCS<br>ICS<br>OCS<br>TCS	3.27<br>3.47<br>3.25<br>3.33

CSR	Clinical indications	Treatment (B; C; A)	Outcome measures	No.of trials (patients): BvC, AvB, AvC	Delta (SE)	TSA scores		ECA scores	
**CD003827**	central venous catheters required	B: Gauze & tape<br>C: Highly Perm TP<br>A: Transparent PU dressing	Catheter related sepsis	2 (115)<br>1 (98)<br>1 (101)	0.077<br>(2.556)	PSS<br>ISS<br>OSS<br>TSS<br>QSS	3.33<br>4.09<br>4.17<br>3.86<br>4.88	PCS<br>ICS<br>OCS<br>TCS	3.33<br>3.47<br>4.17<br>3.66
**CD003842**	Axillary brachial plexus block	B: Multiple injection<br>C: Double injection<br>A: Single injection	Primary anaesthesia failure	4 (270)<br>1 (80)<br>1 (50)	-2.324<br>(1.088)	PSS<br>ISS<br>OSS<br>TSS<br>QSS	4.25<br>4.00<br>4.00<br>4.08<br>5.00	PCS<br>ICS<br>OCS<br>TCS	4.25<br>3.75<br>4.00<br>4.00
**CD003878**	Missing teeth	B: Immediate loading of osseo-integrated implants<br>C: Early loading of osseo-integrated implants<br>A: Conventional	Patients with implant failures	5 (468)<br>8 (310)<br>2 (72)	-0.950<br>(1.651)	PSS<br>ISS<br>OSS<br>TSS<br>QSS	2.75<br>2.90<br>3.84<br>3.16<br>4.38	PCS<br>ICS<br>OCS<br>TCS	2.79<br>2.67<br>3.84<br>3.10
**CD003940**	Oropharyngeal candidiasis	B: Fluconazole<br>C: Clotrimozale<br>A: Itraconazole	Mycological cure	2 (358);<br>3 (400);<br>1 (123)	0.929<br>(0.613)	PSS<br>ISS<br>OSS<br>TSS<br>QSS	3.36<br>4.09<br>2.30<br>3.25<br>3.60	PCS<br>ICS<br>OCS<br>TCS	3.32<br>3.70<br>2.30<br>3.10
**CD004109**	asthma	B: High dose ICS<br>C: Low dose ICS<br>A: Moderate ICS	Morning PEF	2 (282);<br>3 (834);<br>4 (300)	-5.020<br>(12.62)	PSS<br>ISS<br>OSS<br>TSS<br>QSS	3.48<br>3.40<br>4.09<br>3.65<br>4.55	PCS<br>ICS<br>OCS<br>TCS	3.06<br>3.29<br>4.09<br>3.48
**CD004217**	Neonatal circumcision	B; Eutectic mixture of analgesics (EMLA)<br>C: Dorsal penile nerve block (DPNB)<br>A: No treatment/sham	Heart rate -bpm change from baseline (or bmp at endpoint)	1 (29);<br>4 (117);<br>5 (245)	19.680<br>(10.769)	PSS<br>ISS<br>OSS<br>TSS<br>QSS	4.03<br>3.83<br>4.09<br>3.98<br>4.44	PCS<br>ICS<br>OCS<br>TCS	4.03<br>3.97<br>4.09<br>4.03

CSR	Clinical indications	Treatment (B; C; A)	Outcome measures	No.of trials (patients): BvC, AvB, AvC	Delta (SE)	TSA scores		ECA scores	
**CD004291**	organ transplant	B: Fluconazole<br>C: Itraconazole<br>A: Control	Mortality	1 (188);<br>4 (477);<br>2 (191)	-1.046<br>(0.826)	PSS<br>ISS<br>OSS<br>TSS<br>QSS	4.38<br>3.04<br>4.17<br>3.86<br>4.24	PCS<br>ICS<br>OCS<br>TCS	4.38<br>3.29<br>4.09<br>3.92
**CD004293**	nephropathy	B: Alkylating agents<br>C:Steroids<br>A: Placebo/no treatment	ESRF/Death	3 (189);<br>3 (174);<br>3 (333)	0.026<br>(0.886)	PSS<br>ISS<br>OSS<br>TSS<br>QSS	3.90<br>3.79<br>4.67<br>4.12<br>3.38	PCS<br>ICS<br>OCS<br>TCS	3.88<br>3.77<br>4.00<br>3.88
**CD004379**	poor responders to ovarian hyperstimulation	B: GnRH antagonist<br>C: GnRH Flare up protocol<br>A: Conventional GnRHa long protocol	Pregnancy	2 (93);<br>1 (60);<br>1 (54)	-2.468<br>(1.257)	PSS<br>ISS<br>OSS<br>TSS<br>QSS	3.48<br>2.55<br>2.84<br>2.95<br>5.00	PCS<br>ICS<br>OCS<br>TCS	3.48<br>2.36<br>2.84<br>2.89
**CD004386**	afebrile neutropenic patients following chemotherapy	B: Quinolones<br>C: TMP-SMZ (trimethoprim-sulfamethoxole)<br>A: Placebo or no treatment	All cause mortality	10 (917);<br>14 (870);<br>14 (3439)	0.247<br>(0.439)	PSS<br>ISS<br>OSS<br>TSS<br>QSS	3.03<br>4.21<br>4.08<br>3.77<br>2.95	PCS<br>ICS<br>OCS<br>TCS	3.03<br>3.18<br>4.08<br>3.43
**CD004423**	Perioperative complications	B: High volume fluid<br>C: Low volume<br>A: Standard fast	Gastric contents (volume ml)	1 (50);<br>2 (245);<br>8 (522)	-5.408<br>(7.622)	PSS<br>ISS<br>OSS<br>TSS<br>QSS	3.08<br>5.00<br>5.00<br>4.36<br>4.27	PCS<br>ICS<br>OCS<br>TCS	3.00<br>4.47<br>5.00<br>4.16
**CD004610**	diarrhoea	B: Teicoplanin<br>C: Metronidazole<br>A: Vancomycin	Symptomatic Initial Response	1 (59);<br>1 (40);<br>1 (101)	-0.798<br>(1.492)	PSS<br>ISS<br>OSS<br>TSS<br>QSS	3.33<br>5.00<br>4.92<br>4.42<br>4.00	PCS<br>ICS<br>OCS<br>TCS	3.33<br>4.18<br>4.92<br>4.14

CSR	Clinical indications	Treatment (B; C; A)	Outcome measures	No.of trials (patients): BvC, AvB, AvC	Delta (SE)	TSA scores		ECA scores	
**CD004618**	Chronic suppurative otitis media	B: Topical quilone<br>C: Topical non-quinolone<br>A: Topical antiseptic	Discharge at 2-4 weeks	6 (314);<br>2 (430);<br>2 (140)	0.641<br>(0.629)	PSS<br>ISS<br>OSS<br>TSS<br>QSS	3.29<br>3.42<br>4.96<br>3.89<br>3.54	PCS<br>ICS<br>OCS<br>TCS	3.05<br>3.29<br>4.96<br>3.77
**CD004679**	peritoneal dialysis	B: Mupirocin<br>C: Rifampin<br>A: Control	Number of patients with Peritonitis	1 (82);<br>1 (626);<br>1 (64)	-0.685<br>(0.777)	PSS<br>ISS<br>OSS<br>TSS<br>QSS	3.69<br>4.23<br>4.17<br>4.03<br>5.00	PCS<br>ICS<br>OCS<br>TCS	3.38<br>4.13<br>4.17<br>3.89
**CD004756**	acute rejection in kidney transplant	B: Anti-thymocyte globulin (ATG)<br>C: Muromonab-CD3<br>A: Steroid	Failure of reversal of acute first rejection	1 (56);<br>3 (139);<br>1 (120)	-1.664<br>(0.84)	PSS<br>ISS<br>OSS<br>TSS<br>QSS	4.38<br>4.09<br>3.25<br>3.91<br>4.58	PCS<br>ICS<br>OCS<br>TCS	4.38<br>3.82<br>3.25<br>3.82
**CD004785**	meningococcal carrier	B: Ciprofloxacin<br>C: Rifampin<br>A: Placebo	Failure to eradicate (one week follow up)	2 (218);<br>3 (197);<br>6 (725)	3.214<br>(0.871)	PSS<br>ISS<br>OSS<br>TSS<br>QSS	2.88<br>4.88<br>5.00<br>4.25<br>2.18	PCS<br>ICS<br>OCS<br>TCS	2.38<br>4.07<br>5.00<br>3.82
**CD004790**	infants of hepatitis B surface antigen-positive mothers	B: RV<br>C: PDV<br>A: Control	HB event	2 (216);<br>1 (101);<br>3 (272)	-0.007<br>(1.000)	PSS<br>ISS<br>OSS<br>TSS<br>QSS	3.69<br>3.29<br>4.00<br>3.66<br>4.20	PCS<br>ICS<br>OCS<br>TCS	3.63<br>3.94<br>4.00<br>3.86
**CD004861**	oral contraception	B: Levonorgestrel<br>C: Norethindrone (monophasic)<br>A: Gestodene	Discontinuation	2 (1834);<br>2 (817);<br>1 (174)	-0.353<br>(0.599)	PSS<br>ISS<br>OSS<br>TSS<br>QSS	4.38<br>5.00<br>5.00<br>4.79<br>3.88	PCS<br>ICS<br>OCS<br>TCS	4.00<br>4.14<br>4.00<br>4.05

CSR	Clinical indications	Treatment (B; C; A)	Outcome measures	No.of trials (patients): BvC, AvB, AvC	Delta (SE)	TSA scores		ECA scores	
**CD004879**	Preventing influenza in healthy children	B: Live attenuated vaccines (one dose)<br>C: Inactivated vaccines (one dose)<br>A: Placebo	Influenza	2 (222);<br>2 (1646);<br>3 (1361)	-1.495<br>(0.543)	PSS<br>ISS<br>OSS<br>TSS<br>QSS	3.60<br>4.83<br>3.17<br>3.87<br>4.88	PCS<br>ICS<br>OCS<br>TCS	3.60<br>4.57<br>3.17<br>3.78
**CD005015**	kidney transplant recipients	B: Bisphosphonate<br>C: Vit D sterol<br>A: Placebo or no treatment	Change in BMD at lumbar spine	1 (31);<br>3 (150);<br>1 (62)	-2.956<br>(2.295)	PSS<br>ISS<br>OSS<br>TSS<br>QSS	3.13<br>3.29<br>4.05<br>3.49<br>4.17	PCS<br>ICS<br>OCS<br>TCS	3.13<br>2.98<br>3.88<br>3.33
**CD005049**	cardioversion of atrial fibrillation	B: Amiodarone<br>C: Sotalol<br>A: Placebo	Atrial fibrillation recurrence	3 (909);<br>2 (149);<br>7 (1685)	0.910<br>(0.547)	PSS<br>ISS<br>OSS<br>TSS<br>QSS	3.38<br>4.17<br>4.17<br>3.91<br>4.44	PCS<br>ICS<br>OCS<br>TCS	3.38<br>4.22<br>4.17<br>3.92
**CD005115**	Osteoarthritis	B: Rofecoxib 25mg<br>C: Rofecoxib 12.5 mg<br>A: Placebo	Diarrhoea (adverse effect) 6 weeks	4 (1297);<br>3 (550);<br>1 (632)	-1.090<br>(0.739)	PSS<br>ISS<br>OSS<br>TSS<br>QSS	3.96<br>5.00<br>5.00<br>4.65<br>4.78	PCS<br>ICS<br>OCS<br>TCS	3.77<br>5.00<br>5.00<br>4.59
**CD005129**	organ transplant recipients	B: IgC<br>C: CMV IgG<br>A: Placebo/no treatment	No treatment response	1 (18);<br>11 (595);<br>5 (175)	-0.583<br>(1.081)	PSS<br>ISS<br>OSS<br>TSS<br>QSS	3.22<br>4.71<br>3.00<br>3.64<br>4.03	PCS<br>ICS<br>OCS<br>TCS	2.78<br>3.65<br>2.92<br>3.11
**CD005149**	Acute sinusitis	B: Mometasone 400 mcg<br>C: Mometasone 200 mcg<br>A: Placebo	Resolution of symptoms or improved	1 (478);<br>1 (643);<br>1 (95)	-1.590<br>(0.624)	PSS<br>ISS<br>OSS<br>TSS<br>QSS	3.29<br>5.00<br>2.50<br>3.60<br>3.00	PCS<br>ICS<br>OCS<br>TCS	2.75<br>3.75<br>2.50<br>3.00

CSR	Clinical indications	Treatment (B; C; A)	Outcome measures	No.of trials (patients): BvC, AvB, AvC	Delta (SE)	TSA scores		ECA scores	
**CD005347**	contraception	B: MLCu 375<br>C: ML Cu 250<br>A: TCu380A	Pregnancy	2 (1580);<br>3 (6610);<br>1 (1962)	0.927<br>(0.87)	PSS<br>ISS<br>OSS<br>TSS<br>QSS	5.00<br>5.00<br>3.84<br>4.61<br>5.00	PCS<br>ICS<br>OCS<br>TCS	5.00<br>5.00<br>3.84<br>4.61
**CD005351**	Cardiogenic pulmonary oedema	B: Bilevel<br>C: Continmuous positive airway pressure<br>A: Standard medical care	Hopsital mortality	6 (230);<br>9 (501);<br>4 (252)	-0.491<br>(0.772)	PSS<br>ISS<br>OSS<br>TSS<br>QSS	3.34<br>4.00<br>4.00<br>3.78<br>3.87	PCS<br>ICS<br>OCS<br>TCS	3.23<br>3.68<br>4.00<br>3.64
**CD005593**	Alzheimer's disease	B: Donepezil<br>C: Rivastimine<br>A: Placebo	MMSE mean change from baseline (ITT-LOCF)	1 (955);<br>5 (1197);<br>4 (1921)	-0.630<br>(0.747)	PSS<br>ISS<br>OSS<br>TSS<br>QSS	3.79<br>4.84<br>4.09<br>4.24<br>4.48	PCS<br>ICS<br>OCS<br>TCS	3.46<br>4.36<br>4.09<br>3.97
**CD006003**	uncomplicated open cholecystectomy	B: Passive open drain<br>C: Suction drain<br>A: no drain	Wound infection	1 (200);<br>6 (610);<br>4 (873)	-0.263<br>(0.896)	PSS<br>ISS<br>OSS<br>TSS<br>QSS	4.66<br>4.38<br>4.17<br>4.40<br>4.84	PCS<br>ICS<br>OCS<br>TCS	4.41<br>3.04<br>4.17<br>3.87
**CD006257**	diabetic kidney disease	B: AIIRA<br>C: ACEi<br>A: Placebo or no treatment	All cause mortality	1 (250);<br>3 (3251);<br>9 (6781)	-0.181<br>(0.598)	PSS<br>ISS<br>OSS<br>TSS<br>QSS	3.33<br>4.00<br>4.09<br>3.81<br>4.49	PCS<br>ICS<br>OCS<br>TCS	3.31<br>3.47<br>3.59<br>3.45

Table S2: Evidence table for data extraction from trials included in Cochrane systematic reviews (CSRs)

**Author:**								
**Title:**								
**CSR No.**								
**Assessor:**		**Date:**						
**B:**								
**C:**								
**A:**								
**Outcome:**								
**Trial (year)**	**Sample size (n1/n2)**	**Treatment-1**	**Treatment-2**	**Participants**	**Outcome**	**Length of follow up**	**Event rate or value**	**Other notes**
**B vC trials**								
**Sub-total**								
**A v B trials**								
**Sub-total**								

**A v C trials**								
**Sub-total**								

Note: event rate is pooled estimate for direct comparison trials; but the event rate in control group for indirect comparison trials.

Table S3: Sheet for clinical similarity assessment (TSA)

CSR No.	Assessor	Date		B	C	A	Outcome	
	Describe differences between AvB and AvC trials		Applicable? yes-1, no-0	Any important differences?	Is relative effect of a treatment likely to be different because of observed difference between AvB and AvC trials?			
	AvB	AvC			Yes	UC	No	Score
**Participants**								
Age/sex								
Diagnosis/indications								
Severity/baseline risk								
Duration of illness								
Previous treatment failure								
Settings/country								
Other known relative effect moderators								
**Participant similarity score: PSS=**								
**Common control interventions**								
Type of interventions								
Dosages/intensities								
Treatment duration								
Route								
Providers/setting								
Complexity								
Other known relative effect moderators								
**Intervention similarity score: ISS=**								
**Outcome measures**								
Endpoint definition								
Tools/monitoring method/procedures								
Length of follow up								

Other known relative effect moderators								
**Outcome similarity score: OSS=**								
**Average similarity score: TSA = (PSS+ISS+OSS)/3 =**								

Notes – Instructions to assessors for TSA

The assessment of similarity for AIC should be based on the major study characteristics (trial participants, interventions and outcome measures) presented in systematic reviews (study table). This should be based on information collated in Appendix 1, and additional evidence or discussions from the original CSR or primary trials inlcuded in the CSR. Assessment should focus on factors according to original systematic reviews that may possibly affect trial results or generalisibility of trial results. Sample size of trial should be taken into account when there are differences in participants, interventions and outcomes among multiple AvB or among multiple AvC trials.

For each specific field of items (i.e, each row in Appendix 2), assessor first needs to decide whether the specific item is Applicable or Not Applicable. The assessor should mark "1" if Applicable and "0" if Not Applicable. When marked "0" then the score will automatically be locked at 0 and be excluded from the final score calculations in order to avoid giving a higher weight to the not applicable items. If marked "1" then the assessor needs to further decide if there are any important differences between AvB and AvC trials, and whether the relative effect of a treatment might be different because of observed differences between AvB and AvC trials. Specifically, if there are no “other known relative effect modertors” identified, this item should be marked as “not applicable”. If case there is insufficient or missing data, the option of "Yes-missing data" or "No-Missing data" is available.

For each specific field of items (i.e, each row in Appendix 2), the assessors need to make their judgments based on a percentage score. For example if an assessor is unclear for a specific field of item due to some reason but is more inclined to mark either "Yes" or "No" depending on the available evidence then this can be divided as 30% uncertain and 70% No or 70% Yes and vice versa.

If there is any evidence that the pooled relative effect of either AvB or AvC is very likely to be different due to the observed difference between AvB and AvC trials, "Yes" should be selected with a percentage value for your judgement. It can either be 100% Yes if there is substantial evidence or the 100% could be split between "Yes and Unclear" or "Unclear and No" or "Yes, Unclear and No".

Likewise, if it is unclear whether the pooled relative effect of AvB or AvC is affected by the observed difference between AvB and AvC trials, "Uncertain" with a percentage value can be selected. If the assessor is unclear because there is no data available at all then Uncertain column can be marked as 100%. But if the assessor is unclear due to several missing data or other possible treatment moderators that may exist but only in a few trials so that the pooled relative effect may or

may not be significantly affected, then the percentage can be split according to the assessor's judgement. Therefore, the 100% can split between "Unclear and No", "Unclear and Yes" or "Unclear, Yes and No".

If there is no difference between AvB and AvC trials, or the observed difference between them is very unlikely to have any important impact on the pooled relative effect of AvB and AvC, the assessment decision should be "100% “No". This can again be split between "Unclear and No” depending on the assessor's judgement. The judgement can be based on the following situations. (1) There are no important differences between AvB and AvC trials. (2) There is no evidence or any reasons to believe that relative effect of AvB or AvC is associated with the factor or factors that are different between AvB and AvC trials. (3) The relative effect of AvB or AvC may be associated with the factor that are different between trials, but only a very small number of (small) trials were involved and the pooled relative effect is not affected.

The final score of each item that is applicable will be converted from percentage to a score between 0-5 using the equation:

Item similarity score = (Yes% * 0.0 + Unclear% * 2.5 + No% *5.0)/100and

The total score will be the average of each applicable individual score.

Table S4: Sheet for Quality assessment of trials in meta-analysis (QSA)

CSR No.	Assessor	Date					
**B**	**C**	**A**					
**Outcome measured:**							
**Trial (year)**	**Total N**	**Randomisation method (adequate-1; no/uc-0**	**Allocation concealment (yes-1; no/uc -0)**	**Blinding of participants (yes-1; no/uc-0)**	**Blinding of assessor (yes-1, no/uc-0)**	**Dropout (reported and <20% -1; uc or >20% -0)**	**Total**
**BvC trials**							
**Weighted average**							
**AvB trials**							
**Weighted average**							
**AvC trials**							
**Weighted average**							

Note: (1) Rows can be added according to number of trials included. (2) Need to modify cell ranges for the calculation of number of patients, and weighted average, according to number of studies included in each sets.

Instructions to assessors for QSA

The quality of trials in AICic will be assessed based on Jadad's scale, modified according to Schulz's components. Data required to use this scale is usually available from completed CSRs. The quality of individual trials is scored as 1 for adequate and 0 for no or unclear. The quality scores of multiple trials will be weighted by the total number of patients to calculate an average quality score for each of the three sets of trails.

Randomisation method: Select "1" if appropriate method of randomisation described; and "0" if the method was unclear or inappropriate. Appropriate methods of randomisation include: table of random numbers, computer generated, coin tossing, and dice throwing. Examples of inappropriate methods include data of birth, hospital numbers, medical record numbers.

Allocation Concealment: Select "1" if trials reported using either central randomisation, numbered or coded bottles or containers, or a statement indicating that drugs were prepared by a pharmacy. A serially numbered, opaque, sealed envelope is another example of adequate allocation concealment. Select "0" if allocation concealment was unclear or inappropriate.

Blinding of participants: Select "1" if a trial reported that it was "double-blind" or participants were masked about the intervention received or it is a placebo-controlled trial and "0" if patients were not masked.

Blinding of outcome assessor: Select "1" if a trial reported that it was "double-blind" or outcome assessors were masked about the intervention that patients received and select "0" if assessor not masked.

Drop-outs and withdrawals: Select "1" if the number of dropouts reported and <20% and select "0" if number of dropout rates reported and >20% or unclear.

The total quality score for each trial is the number of "1s"

Main references: Moher D, Cook DJ, Jadad Ar, Tugwell P, Moher M, Jones A, et al. Assessing the quality of reports of randomised trials: implications for the conduct of meta-analyses. Health Technol Assess 1999; 3:1-98.

Calculating Quality Similarity Assessment (QSA) score:

Let QT_{AC} and QT_{BC} represent the average quality score for AvC trials and the average quality score for BvC trials respectively. The quality similarity assessment (QSA) score, ranging from 1 (very low) to 5 (very high) was calculated by: $QSA = 5- (|QT_{AC}-QT_{BC}|)$.

Table S5: Sheet for evidence consistency assessment (ECS)

CSR No.	Assessor	Date		B	C	A	Outcome	
	Any differences between DC and AIC trials		Applicable? Yes-1; no-0	Any important differences?	Is relative effect of a treatment likely to be different because of observed difference between trials used in AIC and DC?			
	AIC	DC			Yes	UC	No	Score
**Participants**								
**Age/sex**								
Diagnosis/indications								
Severity/baseline risk								
Duration of illness								
Previous treatment failure								
Settings/country								
Other known relative effect moderators								
**Participant Consistency score: PCS=**								
**Interventions**								
Type of interventions								
Dosages/intensities								
Treatment duration								
Route								
Providers/setting								
Complexity								
Common control in AIC								
Other known relative effect moderators								
**Intervention Consistency score: ICS=**								
**Outcome measures**								
Endpoint definition								

Tools/monitoring method/procedures								
Length of follow up								
Other known relative effect moderators								
**Outcome Consistency score: OCS=**								
**Average Consistency score: ECA = (PCS+ICS+OCS)/3 =**								

Notes - Instruction to assessors for ECA

The assessment of evidence consistency between DC and AIC should be based on the major study characteristics (trial participants, interventions and outcome measures) presented in systematic reviews (study table). This should be based on information collated in Appendix 1, and additional evidence or discussions from the original CSR or primary trials in the CSR. Assessment should focus on factors according to original systematic reviews that may possibly affect trial results or generalisibility of trial results. Sample size of trial should be taken into account when there are differences in participants, interventions and outcomes among multiple trials.

For each specific field of items (i.e, each row in Appendix 3), assessor first needs to decide whether the specific item is Applicable or Not Applicable. The assessor should mark "1" if Applicable and "0" if Not Applicable. When marked "0", the score will automatically be locked at 0 and be excluded from the final score calculations in order to avoid giving a higher weight to the not applicable items. If marked "1" then the assessor needs to further decide if there are any important differences between AIC and DC trials, and whether the relative effect of a treatment might be different because of observed differences between AIC and DC trials. Specifically, if there are no “other known relative effect modertors” identified, this item should be marked as “not applicable”. In case there is insufficient or missing data, the option of "Yes-missing data" or "No-Missing data" is available and should be selected.

For each specific field of items (i.e, each row in Appendix 3), the assessors need to make their judgments based on a percentage score. For example if an assessor is unclear for a specific field of item due to some reason but is more inclined to mark either "Yes" or "No" depending on the available evidence then this can be divided as 40% uncertain and 60% No or 60% Yes and vice versa.

If there is any evidence that the pooled relative effect of either AIC or DC is very likely to be different due to the observed difference between AIC and DC trials, "Yes" should be selected with a percentage value for your judgement. It can either be 100% Yes if there is definite

evidence or the 100% could be split between "Yes and Unclear" or "Yes, Unclear and No".

Likewise, if it is unclear whether the pooled relative effect of AIC or DC is affected by the observed difference between AIC and DC trials, "Uncertain" with a percentage value can be selected. If the assessor is unclear because there is no data available at all then Uncertain column can be marked as 100%. But if the assessor is unclear due to several missing data or other possible treatment moderators that may exist but only in a few trials so that the pooled relative effect may or may not be significantly affected, then the percentage can be split according to the assessor's judgement. Therefore, the 100% could be split between "Unclear and No", "Unclear and Yes" or "Unclear, Yes and No". Please not that the Intervention Consistency Score (ICS) in the Appendix 3 could be higher than the Intervention Similarity Score (ISS) in Appendix 2 due to 8 items instead of only 7.

If there is no difference between AIC and DC trials, or the observed difference between them is very unlikely to have any important impact on the pooled relative effect of AIC and DC, the assessment decision could be "100% No". This can again be split between "Unclear and No", "Unclear and Yes" or "Unclear, Yes and No" depending on the assessor's judgement. The judgement can be based on the following situations. (1) There are no important differences between AIC and DC trials. (2) There is no evidence or any reasons to believe that relative effect of AIC or DC is associated with the factor or factors that are different between AIC and DC trials. (3) The relative effect of AIC or DC may be associated with the factor that is different between trials, but only a very small number of (small) trials were involved and the pooled relative effect is not affected.

In principle, the total consistency score should be equal to or lower than the similarity score.

The final score of each item that is applicable will be converted from percentage to a score between 0-5 and the total score will be the average of each applicable individual score.

Table S6: Evidence table – topical azelaic acid vs. topical metronidazole for rosacea (case study CD003262)

**Author:**	van Zuuren							
**Title:**	Interventions for rosacea							
**CSR No.**	CD003262							
**Assessor:**	SP/TX	**Date:**	30/11/2009					
**B:**	Topical azelaic acid							
**C:**	Topical metronidazole							
**A:**	Placebo							
**Outcome:**	Physicians global evaluation of improvement							
**Trial (year)**	**Sample size (n1/n2)**	**Treatment-1**	**Treatment-2**	**Participants**	**Outcome**	**Length of follow up**	**Event rate or value**	**Other**
**BvC trials**								
Elewski 2003	124/127	Azelaic acid<br>15% gel x 2 times daily<br>Duration = 15 weeks	Metronidazole<br>0.75% gel x 2 times a day	Setting: Multicentre, USA<br>N = 251; Age = 46 & 49 (mean); Sex = 66M/185F<br>Patients with papulopustular rosacea with persistent erythema and telangiectasia. 10-15 inflammed facial papules.<br>Excluded mild rosacea, marked ocular manifestations, hypersensitivity, lactating mothers.	Investigators global assessment - 7 point static scoring system	15 weeks (baseline every 4 weeks)	62.2%	
**Sub-total**	124/127	See above	See above	See above	See above	See above	62.2%	
**A v B trials**								
Bjerke 1999	76/38	Azelaic acid<br>20% cream x 2 times daily<br>Duration = 12 weeks	Placebo<br>2 times daily	Setting: Multicentre, Norway<br>N = 116; Age = 48.4 & 50.3 (mean); Sex = 55M/59F<br>Patients with papulopustular rosacea.<br>Excluded any ocular involvement.	Physician's global impression of improvement	12 weeks	55.3%	
Thiboutot 2003a	164/165	Azelaic acid<br>15% gel x 2 times daily<br>Duration = 12 weeks	Placebo<br>2 times daily	Setting: Multicentre, USA<br>N = 329; Age = 48 & 49 (mean); Sex = 84M/245F<br>Patients with papulopustular rosacea (moderate), 8-50 inflammed facial papules.<br>Excluded patients with marked ocular involvement, hypersensitivity.	Investigator's global assessment - 7 point static scoring system.	12 weeks	47.9%	

Thiboutot 2003b	169/166	Azelaic acid<br>15% gel x 2 times daily<br>Duration = 12 weeks	Placebo<br>2 times daily	Setting: Multicentre, USA<br>N = 335; Age = 47 & 48 (mean); Sex = 93M/242F.<br>Patients with papulopustular rosacea (moderate), 8-50 inflammed facial papules.<br>Excluded patients with marked ocular involvement, hypersensitivity.	Investigator's global assessment - 7 point static scoring system.	12 weeks	54.8%	
**Sub-total**	409/369	Azelaic acid<br>15% gel or 20% cream applied 2 times a day<br>Duration = 12 weeks	Placebo<br>2 times daily	Setting: Multicentre, Norway, USA<br>Age = 48 (mean); Sex = >%F<br>Patients with papulopustular rosacea.<br>Excluded ocular involvement.	Investigator's global assessment - 7 point static scoring system	12 weeks	51.8%	
**A v C trials**								
Bjerke 1989b	50/47	Metronidazole<br>1% cream x 2 times daily<br>Duration = 8 weeks	Placebo<br>2 times daily	Setting: Multicentre, Norway<br>N = 97; Age = 47 (mean); Sex = 44M/53F<br>Patients with papulopustular rosacea, erythema and telangiectasia (10 papules).<br>Excluded ocular involvement, pregnancy, lactation, treatment with antibiotics.	Physician's global evaluation	8 weeks	55.3%	
Breneman 1998	89/50	Metronidazole<br>1% cream once daily<br>Duration = 10 weeks	Placebo	Setting: Multicentre, USA<br>N = 156; Age = 48.5 (mean); Sex = 51M/105F<br>Patients with papulopustular rosacea.	Physician's global evaluation	10 weeks	2.0%	
Nielsen 1983a	40/37	Metronidazole<br>1%cream x 4 times daily<br>Duration = 8 weeks	Placebo<br>4 times daily	Setting: Single, Sweden<br>N = 81; Age = 47 (mean); Sex = 32M/49F<br>Patients with rosacea of differing degrees of severity.	Physician's global evaluation	8 weeks	21.6%	
**Sub-total**	179/134	Metronidazole<br>1% cream once daily or 2 times or 4 times daily.<br>Duration = 8 or 10 weeks	Placebo<br>Duration = 8 or 10 weeks	Setting: Single or Multicentre, Sweden, Norway, USA<br>Age = 47 (mean); Sex = >%F.<br>Patients with papulopustular rosacea or rosacea of differing degrees of severity.	Physician's global evaluation	8 or 10 weeks	26.1%	

Table S7: Clinical similarity assessment results – case study (CD003262)

Assessment items	Important differences between AvB and AvC trials?	Effect modification? Yes/Uncertain/No	Score
**Participants**			
Age/sex	No	0% / 0% / 100%	5.0
Diagnosis/indications	No	0% / 0% / 100%	5.0
Severity/baseline risk	Event rate in placebo arms: 48.2% vs. 73.9%	R1: 0% / 100% / 0%<br>R2 20% / 80% / 0%	R1: 2.5<br>R2: 2.0
Duration of illness	Missing data	0% / 100% / 0%	2.5
Previous treatment failure	Missing data	0% / 100% / 0%	2.5
Settings/country	Norway or Germany vs. Norway or USA or Sweden	R1: 0% / 20% /80%<br>R2: 0% / 0% / 100%	R1: 4.5<br>R2: 5.0
Other effect moderators	Not identified		
**Participant similarity**			**3.67**
**Common control interventions**			
Type of interventions	No	0% / 0% / 100%	5.0
Dosages/intensities	2 times daily vs. 1 or 2 or 4 times daily	R1: 0% / 20% / 80%<br>R2: 0% / 50% / 50%	R1: 4.5<br>R2: 3.75
Treatment duration	12 weeks vs. 8 or 10 weeks	R1: 0% / 20% / 80%<br>R2: 0% / 50% / 50%	R1: 4.5<br>R2: 3.75
Route	No	0% / 0% / 100%	5.0
Providers/setting	No	0% / 0% / 100%	5.0
Complexity	No	0% / 0% / 100%	5.0
Other effect moderators	Not identified		
**Intervention similarity**			**R1: 4.83**<br>**R2: 4.58**
**Outcome measures**			
Endpoint definition	No	0% / 0% / 100%	5.0
Tools/method/procedures	7 point static scoring or not specified vs. not specified	R1: 0% / 100% / 0%<br>R2: 20% / 80% / 0%	R1: 2.5<br>R2: 2.0
Length of follow up	12 weeks vs. 8 or 10 weeks	R1: 0% / 100% / 0%<br>R2: 20% / 80% / 0%	R1: 2.5<br>R2: 2.0
Other effect moderators	Not identified		
**Outcome similarity**			**R1: 3.33**<br>**R2: 3.00**
**Overall TSA**			R1: 3.94<br>R2: 3.75<br>**Av: 3.85**

Note: R1 refers to reviewer 1, R2 to reviewer 2, and Av refers to average. Differences between the two assessors may remain after discussion.

Table S8: ECA- evidence consistency assessment results –case study (CD003262)

Assessment items	Important differences between AIC and DC trials?	Effect modification? Yes/Uncertain/No	Score
**Participants**			
Age/sex	No	0% / 0% / 100%	5.0
Diagnosis/indications	No	0% / 0% / 100%	5.0
Severity/baseline risk	Event rate in placebo arms: 48.2% vs. 73.9%.<br>Event rate in active drug arms: AIC 28.9% and 49.7% versus DC 30.7% and 44.9% respectively	R1: 0% / 100% / 0%<br>R2: 20% / 80% / 0%	R1: 2.5<br>R2: 2.0
Duration of illness	Missing data	0% / 100% / 0%	2.5
Previous treatment failure	Missing data	0% / 100% / 0%	2.5
Settings/country	AIC: Norway or Germany or USA or Sweden.<br>DC: USA	R1: 0% / 20% / 80%<br>R2: 0% / 0% / 100%	R1: 4.5<br>R2: 5.0
Other effect moderators	Not identified		
**Participant consistency**			**3.67**
**Interventions (A vs B)**			
Type of interventions	No	0% / 0% / 100%	5.0
Dosages/intensities	AIC: TAA 20% or 15% x2 daily vs. TM cream 1% x1 or 2 or 4 daily.<br>DC: TAA newly developed gel 15% x2 daily vs. TM gel 0.75% x2 daily	20% / 80% / 0%	2.0
Treatment duration	AIC: 8 or 10 or 12 weeks.<br>DC: 15 weeks.	20% / 80% / 0%	2.0
Route	No	0% / 0% / 100%	5.0
Providers/setting	No	0% / 0% / 100%	5.0
Complexity	No	0% / 0% / 100%	5.0
Common control in AIC	Placebo 12 weeks vs. 8 or 10 weeks	R1: 0% / 0% / 100%<br>R2: 0% / 50% / 50%	R1: 5.0<br>R2: 3.75
Other effect moderators	Not identified		
**Intervention consistency**			**R1: 4.14**<br>**R2: 3.96**
**Outcome measures**			
Endpoint definition	No	0% / 0% / 100%	5.0
Tools/method/procedures	AIC 7 point static scoring or not specified vs. DC 7 point scoring	R1: 0% / 100% / 0%<br>R2: 20% / 80% / 0%	R1: 2.5<br>R2: 2.0
Length of follow up	AIC 8 or 10 or 12 weeks vs. DC 15 weeks.	R1: 0% / 100% / 0%<br>R2: 20% / 80% / 0%	R1: 2.5<br>R2: 2.0
Other effect moderators	Not identified		
**Outcome consistency**			**R1: 3.33**<br>**R2: 3.00**
**Overall ECS**			**R1: 3.71**<br>**R2: 3.54**<br>**Av: 3.63**

Note: TAA – topical azelain acid. TM – topical metronidazole. R1 - Reviewer 1; R2 - Reviewer 2; Av - Average. Difference between the two assessors may remain after discussion.

Table S9: Assessment of quality of studies included – case study (CD003262)

Trial (year)	Total N	Randomisation method (adequate-1; no/unclear-0	Allocation concealment (yes-1; no/unclear -0)	Blinding of participants (yes-1; no/unclear-0)	Blinding of assessor (yes-1, no/unclear-0)	Dropout (reported and <20% -1; unclear or >20% -0)	Total
**BvC trials**							
Elewski 2003	251	1	1	1	1	1	5
**Weighted average**	**251**						**5.000**
**A v B trials**							
Bjerke 1999	114	0	0	1	1	1	3
Thiboutot 2003a	329	1	1	1	1	1	5
Thiboutot 2003b	335	1	1	1	1	1	5
**Weighted average**	**778**						**4.707**
**A v C trials**							
Bjerke 1989b	97	1	1	1	1	1	5
Breneman 1998	139	0	0	1	1	1	3
Nielsen 1983a	77	1	1	1	1	1	5
**Weighted average**	**313**						**4.112**
**QSA score = 5 – ABS(QAC – QBC) =**							**4.405**
